# A multi-omics dataset of the response to early plant polysaccharide ingestion in rabbits

**DOI:** 10.1038/s41597-024-03471-1

**Published:** 2024-06-25

**Authors:** Charlotte Paës, Martin Beaumont, Thierry Gidenne, Karine Bébin, Joël Duperray, Charly Gohier, Emeline Guené-Grand, Gwénaël Rebours, Adrien Castinel, Céline Barilly, Béatrice Gabinaud, Carole Bannelier, Laure Gress, François Laperruque, Patrick Aymard, Anne-Marie Debrusse, Laurent Cauquil, Géraldine Pascal, Sylvie Combes

**Affiliations:** 1 GenPhySE, Université de Toulouse, INRAE, ENVT, 31326 Paris, Castanet-Tolosan France; 2 CCPA, ZA du Bois de Teillay, 35150 Janzé, France; 3 INP-Purpan, Toulouse, 31076, France; 4 EVIALIS, Lieu dit Talhouët, 56250 Saint Nolff, France; 5 MiXscience, 2 avenue de Ker Lann, 35170 Bruz, France; 6 WISIUM, Rue de l’église, BP50019, 02407 Chierry, France; 7 TECHNA, Route de St-Étienne-de-Montluc, 44220 Couëron, France; 8 GeT‐PlaGe, Genotoul, INRAE, Castanet-Tolosan, 31326 France

**Keywords:** Feeding behaviour, Microbiome, Antibodies

## Abstract

The transition from a milk-based diet to exclusive solid feeding deeply modifies microbiota-host crosstalk. Specifically, early ingestion of plant polysaccharides would be one of the main nutritional components to drive host-microbiota-interaction. To capture the effects of polysaccharides early-life nutrition (starch *vs* rapidly fermentable fiber) on the holobiont development, we investigated on the one hand the gut bacteriome and metabolome and on the other hand the transcriptome of two host gut tissues. Rabbit model was used to study post-natal co-development of the gut microbiota and its host around weaning transition. The assessment of the microbial composition of the gut appendix together with the caecum was provided for the first time. Gene expression signatures were analyzed along the gut (ileum and caecum) through high-throughput qPCR. The data collected were completed by the analysis of animal growth changes and time-series assessment of blood biomarkers. Those accessible and reusable data could help highlight the gut development dynamics as well as biological adaptation processes at the onset of solid feeding.

## Background & Summary

The digestive microbiota of mammals represents an ecological community of microorganisms that resides in the gastrointestinal tract and acts as a symbiotic partner through a dynamic crosstalk with the host, affecting its digestive and immune functions^1–3^. While the holobiont is still in construction, the introduction of solid food in the diet reshapes the gut microbiome through the provision of new nutritional substrates resistant to host digestion^4–6^. The influx of such new nutrients directly modulates the host physiology as well^7^. If this diet transition is recognized as a major step in the young’s development, the mechanistic understanding of the changes occurring in the gut and the long-term consequences of pre-weaning experiences remain unknown. A multi-level approach, from the molecular to the animal levels, with a dynamic follow-up appears necessary to capture the process occurring at the suckling-weaning transition.

In this study, three conditions at the onset of solid food ingestion were tested to investigate the effects of 1/ the timing of solid food introduction and 2/ dietary polysaccharides content on the co-maturation of rabbits and their microbiota. In classical breeding systems, solid food is reachable for suckling rabbits as of 16–18 days of age^8^. Following the feeding behaviour of wild rabbits in the nest, we investigated the effects of an earlier access to solid substrates, available from 3 days of age. Two polysaccharide contents differing by their levels of rapidly fermentable fibers and starch were tested to assess the effects of these two major components of solid-based diets on the gut bacteriome. Rabbit model was used since it is a monogastric species that strongly relies on gut homeostasis for its health and has been successfully used for modeling infections with enteric pathogens^9,10^. Besides, rabbits present a specific behaviour pattern allowing mother-offspring separation most of the time. Easy control of early-life ingestion (milk and food) can therefore be performed to investigate post-natal food ingestion apart from nursing influence. Last but not least, a better understanding of the multidimensional aspects of gut health in rabbits appears essential to improve rabbit breeding practices.

**Fig. 1:**
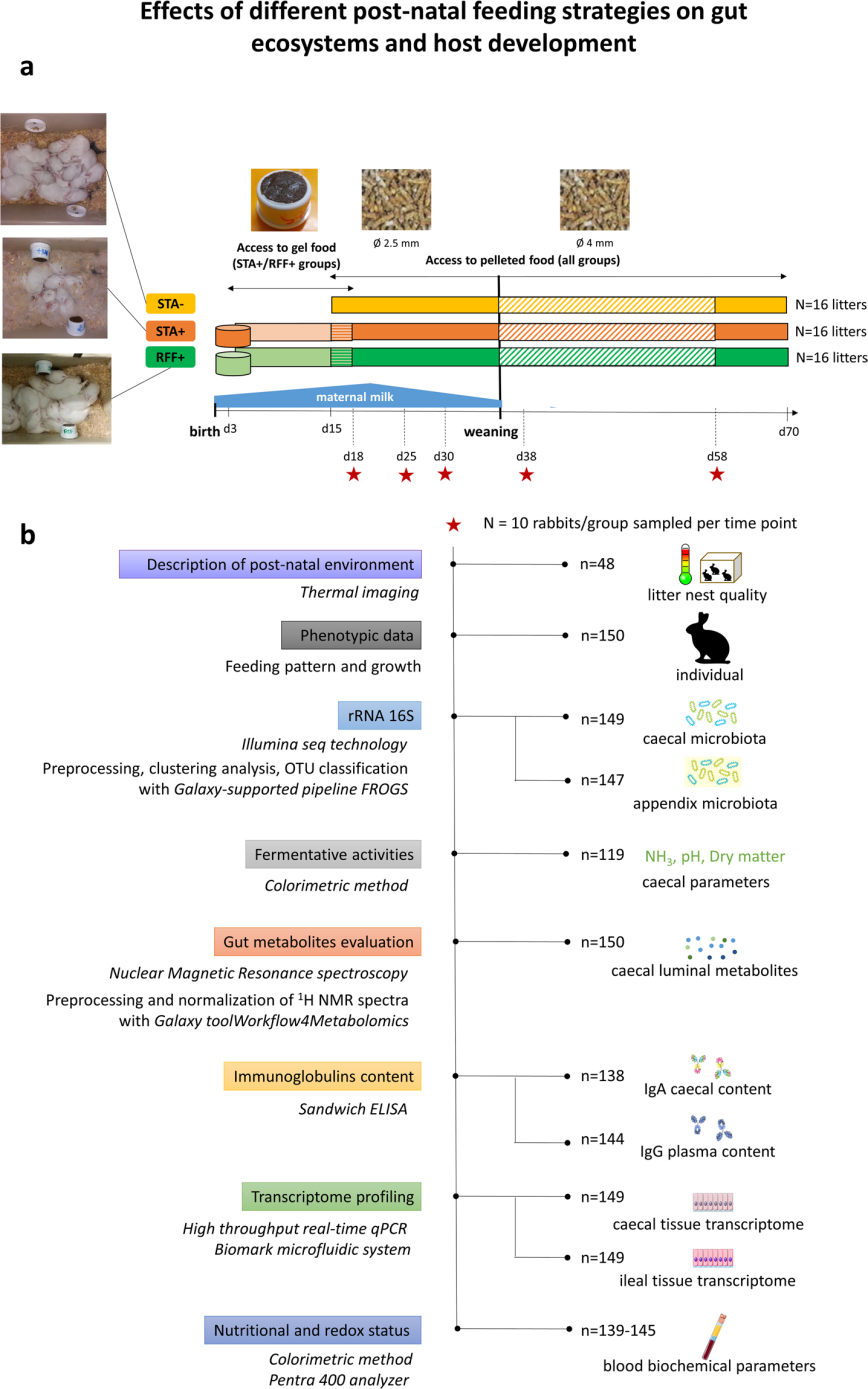
Experimental design (**a**) and data collection (**b**). This study aimed to provide insights into host and gut microbiota responses to changes in the timing of solid food introduction (groups STA-/STA+) when two different diets were provided throughout the life (diets STA and RFF). STA-: access to starch-enriched food in a pellet form from 15 to 70 days-old. STA+: access to starch-enriched food from 3 to 18 days-old in a gel form and from 15 to 70 days-old in a pellet form. RFF+: access to food rich in rapidly fermentable fibers from 3 to 18 days-old in a gel form and from 15 to 70 days-old in a pellet form.

In this data descriptor, we present time-series data collected at the weaning transition (Fig. 1). Information regarding the growth of rabbits and their feeding behavior were included. At an individual-based level, we provide information relative to gut communities (16S ADNr) and their activities (luminal metabolome) together with rabbit gene expression (high-throughput qPCR) to uncover some aspects of host/microbiota co-maturation that cannot be fully understood with a unique data-type. We report data on 150 rabbits sampled from 30 litters, giving access to information regarding the proportions of 1 197 taxa in the appendix (lymphoid organ) and caecal microbiota, the levels of 29 luminal metabolites and the expression of 48 transcripts of two rabbit gut tissues. Quantification of caecal IgA, plasma IgG and serum redox parameters complement those data. We aimed to generate a comprehensive and combined multi-omics dataset with samplings distributed at 5 different dates, corresponding to the critical phases of food transition^11^. We intended to follow FAIR principles by providing machine-readable and detailed metadata. Data collection, analysis strategies and corresponding repositories are detailed in this data descriptor to enable a human-friendly data mining.

A part of these datasets were previously disclosed in our associated results paper^12^, with this work intended to provide further methods descriptions and validation to facilitate re-use. New data are presented compared to our previous publication, e.g., biochemical measurements of blood content, technical replicates for 16S rDNA sequencing, control strains characterization, rabbit nest quality evaluation and ileal gene expression.

In our companion paper^12^, we demonstrated that the ingestion of small quantity of solid food in early life while maintaining milking could effectively accelerate gut microbiota maturation, with spatial-specific effects in the gut. Increasing the ingestion of rapidly fermentable fibers contributed to a specialization of the microbiota towards fibers-degradation activities. Effects of those strategies on the gut transcriptome and the host phenotype were limited in a context of good sanitation facilities. In our results paper, data analysis was restricted to single-omics approach.

Time-monitoring of gut bacterial communities with associated host responses can allow researchers to extract a better picture of early-life developmental process. This comprehensive and reusable collection of data can be further explored by integrating the different types of datasets provided (multi-omics approaches). This could help revel some interplay mechanisms. To our knowledge, some data were generated for the first time with this experiment, thus providing new information to scientific community (e.g. investigation of caecal and appendix microbiomes in the same individuals or evaluation of plasmatic fatty acids and protein contents in rabbits at different stages). Controls for technical and biological reproductibility of the data were generated, thus leading to valuable data resources for microbiologists and physiologists. We provided herein the description of the methods used together with raw and processed data to ensure easy sharing and re-processing thanks to analysis script provided on the Github platform.

## Methods

Rabbits breeding and feeding, sampling conditions, biological acquisition and bioinformatics pipelines (including amplicon sequencing and sequence-based microbiota analysis) were previously disclosed in our associated results paper^12^. Detailed procedures or additional information such as complete lab steps for performing NMR measurements and RNA extraction are provided here to ensure that our datasets are understandable and reusable.

### Animal management and housing

The animal husbandry procedure is described in our previous article^12^. In this data descriptor, the cages used for the experiment are described in more detail, as the housing system was specifically designed for this study. Additional data collection on nest quality is also presented.

**Fig. 2:**
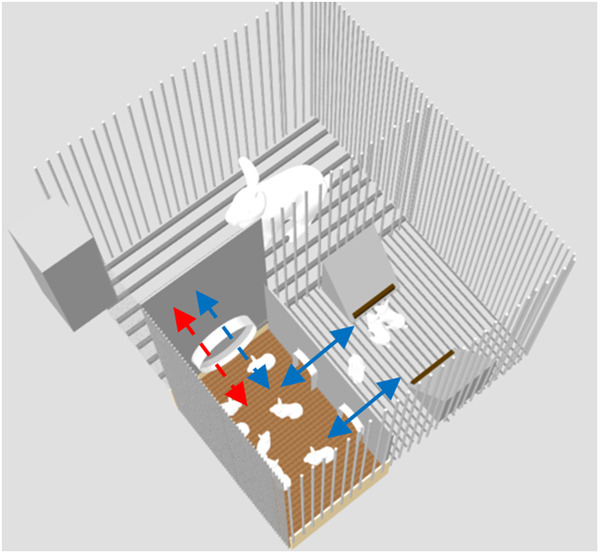
3D model of the experimental housing system used before weaning. View from the top of a nursing cage where the doe and its kits are raised together for 35 days. Blue arrows represent possible movements of the kits while red arrow represents possible path for the doe. Until day 21, the nest was only opened once a day for suckling (dottled arrows).

Forty-eight crossbred litters (Hyplus PS19 × Hyplus PS59) were raised with their doe in wire cages (width: 62 × length: 69 × height: 62 cm) equipped with nests for the pups (width: 25 cm; length: 38 cm; height: 20 cm). Starter food gels were produced daily and were provided to suckling rabbits directly in the nest inside plastic cups (30 mL; height: 32 mm; diameter: 40 mm; GOSSELIN®, Le Mans, France). Cages were designed to allow the mother and its litter to be fed separately (Fig. 2). In that respect, a wire mesh partition separated the doe from the area containing the kits’ feeders. The doe’s feeder in the cage was elevated so that the kits could not access to the doe’s feed. The day following parturition, the thermal quality of the nests was evaluated using a thermal infrared camera (Fluke Ti450, Everett, Wash, USA). After nest quality assessment, litter size was standardized to 10 kits. At 3 days, litters were allocated to the three experimental groups according to their body weight (kit weight: 99 ± 9 g) and the doe’s parity (5 ± 2 parturitions) (Fig. 1a).

### Experimental design

Two diets were formulated for the experiment. They differed only in the ratio of rapidly fermentable fibers (RFF)/starch (STA) (0.6 for STA diet and 2.0 for RFF diet). Detailed diet formulations are provided in our companion paper, together with the procedure for feed distribution^12^.

### In-farm measurements

We provide herein more detailed information on the collection of performance data (feed ingestion and animal weight). The ingestion of starter food gel was measured daily from 7 days (start of significant ingestion) by the difference in weight between gel offered and gel left. Individual gel ingestion was obtained after correction for gel water loss and final division by the number of kits in the nest. Water loss was estimated inside breeding rooms in empty nests (n=3 measurements/day for each gel type) and averaged to 8% of the initial gel weight. The dry matter content of the starter feed gels was 26% and 89% in the pellets. Milk consumption was measured by weighing the rabbit doe before and after nursing on 3, 7, 10, 14, 17 and 21 days^13^. Pellet ingestion before weaning was assessed at 18, 21, 25, 28, 32 and 36 days of age. Mortality was registered daily and growth was assessed before weaning by weighing the litters at 3, 14, 21 and 28 days of age. After weaning, individual rabbit weights were recorded at 36, 50, 64 and 71 days of age. Live body weights were recorded automatically using a scale (SWR08-10S Plateforme 310 × 275 Trolley, Balea, Saint Mathieu de Tréviers, France) connected to an automatic recording system (Teo or AGPA, Balea, Saint Mathieu de Tréviers, France) with a Bluetooth connection^14,15^.

### Killings and sampling

Ten healthy pups per group (one pup per litter) were selected the days of sampling (n=150 pups euthanized in total). Description of the criteria and procedure for euthanasia are detailed below to supplement the methods provided before^12^.

The sampling dates were chosen based on the feed transition process^11^:

- 18 days of age (solid food consumption remains minor)
- 25 days (dietary switch with marked increase of solid food ingestion proportion compared to milk)
- 30 days (solid-based diet with low amounts of milk consumed)
- 38 days (beginning of exclusive solid diet related with high susceptibility to digestive diseases)
- 58 days (settled exclusive solid feeding with more stable sanitary status)

At days 18, pups that exhibited interest for gel food were preferably selected while at days 25, 30, 38 and 58, the pups were randomly chosen. Before weaning, the killing procedure was performed 1 to 2 hours after suckling, while after weaning the rabbits were sampled 2 to 3 hours after feed distribution to obtain similar postprandial state. After the determination of sex and body weight of the animals chosen, they were killed by electronarcosis and exsanguination.

Blood was collected at exsanguination in EDTA and dry tubes for plasma and serum preparation respectively. EDTA tubes were immediately put on ice until centrifugation (800 g for 10 min at 4 °C). After collection of 2.5 mL of blood, dry tubes were let undisturbed at room temperature for 20 minutes. The clot was then removed by centrifugation (1 800 g for 10 min in a regular centrifuge). Resulting supernatants were collected and stored at −20 °C. After isolation of the caecum and the appendix vermiformis, they were weighted (OHAUS scale, Parsippany, NJ, USA) and up to 500 mg of luminal contents were collected in sterile tubes stored at −80 °C until the extraction step. Between samples, protective gloves were replaced and the material was sterilized to prevent crossed contamination. 0.5 cm of tissues from proximal caecum and distal ileon were quickly extracted at 3 cm up and down the *Sacculus Rototondus*. After washing in ice-cold PBS, they were immediately snap-frozen in liquid nitrogen and stored at −80 °C prior to further processing. 1 g of caecal content was collected and diluted in H_2_SO_4_ (at 2% w/v) to quantify ammoniac (NH_3_) concentrations at days 25, 30, 38 and 58 (insufficient quantities at day 18). Caecal pH was measured after previous samplings by introducing a glass electrode at the ileocecal junction (VWR Collection SP225, Radnor, Pennsylvanie, USA). Finally, around 2 g of caecal contents were collected to determine dry matter level after heating at 103 °C for 24 h.

### Metabolomics

Caecal contents (100 mg) were homogenized in 500 µL phosphate buffer (sodium phosphate 0.2 M, pH 7.4, trimethylsilylpropanoic acid 1 mmol/L, 80% deuterated water, and 20% water) in 2 mL FastPrep tubes (Lysing D matrix) by using a FastPrep Instrument (MP biomedicals, Irvine, CA). After centrifugation (12 000 g, 4 °C, 10 min), the supernatant was transferred to a new tube. The pellet was resuspended in 500 µL phosphate buffer and the homogenization and centrifugation steps were repeated. Supernatants from both extraction steps were pooled and centrifuged twice to remove particles (18 000 g, 30 min, 4 °C). The resulting supernatants (600 µL) were transferred to 5 mm NMR tubes. NMR spectra were obtained with an Avance III HD NMR spectrometer operating at 600.13 MHz for 1H resonance frequency using a 5 mm inverse detection CryoProbe (Bruker Biospin, Rheinstetten, Germany) in the MetaboHUB-MetaToul-AXIOM metabolomics platform (Toulouse, France). The 1H NMR spectra were acquired at 300 K using the Carr-Purcell-Meiboom-Gill (CPMG) spin-echo pulse sequence with presaturation. A total of 128 scans (16 dummy scans) were collected in 32 K data points using a spectral width of 20 ppm and an acquisition time of 1.36 s.

Pre-processing of the spectra (group delay correction, solvent suppression, apodization with a line broadening of 0.3 Hz, Fourier transform, zero order phase correction, shift referencing on TSP, baseline correction, setting of negative values to zero) was performed in the Galaxy tool Workflow4Metabolomics following guidelines^16^. After water region (4.5–5.1 ppm) exclusion, spectra (0.5–9 ppm) were bucketed (0.01 ppm bucket width) and normalized by sample weight in Workflow4Metabolomics. Representative samples were characterized by 2D NMR experiments (1H-1H COSY and 13C-1H HSQC). For metabolite identification, 1D and 2D NMR spectra of pure compounds prepared in the same buffer and acquired with the same spectrometer were overlayed with samples spectra. For each metabolite identified (n = 29), one bucket non-overlapping with other metabolites was selected and its value (area under the curve of the 0.01 ppm segment, normalized to the sample weight) was used for quantification. The chemical shift of buckets selected for quantification of each metabolite is indicated in supplemental table S1.

### Gene expression profiling

To improve the reproducibility of our work, we provide below new information on our RT-qPCR protocol.

Caecal (n = 149) and ileal (n = 149) tissues were homogenized in 800 μL TRI reagent (ZymoResearch) with one sterile stainless steel 5 mm diameter bead (Qiagen, Hilden, Germany) by using a TissueLyzer (Qiagen) with two 3 min cycles at 30 Hz. After centrifugation (12 000 g, 4 °C, 10 min), 300 μL of supernatant was used for RNA extraction by using Direct-zol kit (ZymoResearch) following the manufacturer instruction, including a DNAse I treatment with DNA digestion buffer. RNA concentration and quality (260:280 and 260:230 ratios) were analyzed with NanoDrop 8000 (Thermo Fisher Scientific). cDNA were prepared from 1 μg RNA, 1 μL Oligo(dT) (100 µM) and 1 μL dNTP Mix (10 mM each) (Promega, Madison, WISC, USA). After heating the mixture to 65 °C for 5 min and quick chill on ice, the contents of the tube was mixed with 4 μL 5X First-Strand Buffer (Invitrogen, ThermoFisher Scientific), 2 μL 0.1 M DTT, 1 μLSuperscript II Reverse Transcriptase (ThermoFisher Scientific) and 1 μL RNasin®Ribonuclease Inhibitor (Promega). After incubation (42 °C for 60 min), the reaction was inactivated by heating at 70 °C for 15 min. cDNA synthesis was checked with the house keeping gene *GAPDH* by using real-time quantitative PCR performed in QuantStudio 6 (Thermo Fisher Scientific, Waltham, MA, USA) with a reaction solution containing 2.5 μl of SybrGreen fluorescent DNA-binding dye (ThermoFisher Scientific), 0.2 μL of each primer at 10 µM, 0.9 μM of sterile water and 1 µL of DNA diluted at 1:10. We performed 40 PCR amplification cycles with an annealing temperature of 60 °C. After this control, high throughput real-time qPCR was performed for each tissue using the Biomark microfluidics system using a 96.96 Dynamic Array™ IFC for gene expression (Fluidigm, San Francisco, CA, USA). 1.3 μL of cDNA (5 ng.μl^−1^) was added to the array and processed on the fluidics system according to the guidelines of the GENOTOUL platform. Briefly, an initial high temperature activation step of 10 minutes was performed into the PCR reaction with 35 amplification cycles at 60 °C later on. The sequences of the primers used are presented in our companion paper. Genes with suitable melt curve aspect, good efficiency (>90% and < 110%) and linearity were kept for statistical analysis. *GAPDH* and *TOP1* gene were selected as housekeeping genes for caecal and ileal tissue respectively, based on their stability over time and between groups, in order to calculate gene expression using the 2^−ΔΔCt^ method.

### Enzyme-linked immunosorbent assay (ELISA) measurements

The ELISA laboratory procedure has been shortly described in our companion document^12^. Further details, such as the construction of the calibration curve, are given below for better reproducibility.

In order to extract the immunoglobulins A (IgA) from the caecal content, 200–500 mg of digesta was diluted at 50 mg/mL in cold TBS. After shaking the solution thoroughly, and centrifugation at 3000 g for 10 min at 4 °C, the supernatant was collected for measurement. Total plasma IgG or caecal content IgA levels were determined in duplicates by sandwich ELISA in 96-well plates coated with specific polyclonal goat anti-rabbit IgG or IgA antibody (Bethyl Laboratories, Montgomery, Texas, USA) with further plate reading at 450 nm after fixation of the reaction between HRP conjugated antibodies (Bethyl Laboratories) and TMB. IgG were quantified using a reference IgG serum (Bethyl Laboratories). Regarding IgA, 12 samples were pooled to build a reference sample for the standard curve construction. For both measurements, seven calibrator points and water blank were added in duplicate to the microwell plates along with the samples. While linear standard curves were sufficient to fit the seven calibrators used for IgG quantification in plasma, a 4-parametric logistic model (4-PL) was used to improve the fit of caecal IgA calibration curve (average R^2^ with the 7 calibrators points = 0.98).

### Biochemical evaluation of blood nutritional and redox status

26 µL of plasma per sample were collected for the dosage of free fatty acids, triglycerides and total protein. After storage at −20 °C, the dosages were performed with Pentra 400 device (HORIBA Medical, Grabels, France) at the Anexplo Phenotypage GENOTOUL platform (Toulouse, France). One sample (ID: 334) was diluted at 1:30 to fit the absorbance linearity range.

### Data analysis

One analytical workflow using R version 4.0.0 is shared publicly in the repository https://github.com/paescharlotte/early_life_nutrition_rabbit^17^.

## Data Records

A summary of all the data collected during this experiment and their repository access are given in Table 1 and Fig. 1b.

**Table 1:** Accessibility of the public data sets provided.

Data set	Database	Accession
Thermal images used to assess nest quality at day 1	Data INRAE, “J1_1802_cagenumber.jpg”	10.15454/QSTXWF
Feeding pattern before weaning of all the rabbits raised during the experiment	Data INRAE, “Milk_consumption.csv”, “Gel_consumption.csv”, “Pellet_consumption.csv”, “Gel_consumption.mp4” and “Gel_consumption_access.mp4”	10.15454/QSTXWF
Growth data before and after weaning of all the rabbits raised during the experiment	Data INRAE, “Rabbits_weight.xlsx”	10.15454/QSTXWF
Phenotypic characterization of the rabbits sampled	Data INRAE, “Host_data.csv”	10.15454/QSTXWF
Transcript profiling	Data INRAE, “RNA_caecum.csv” and “RNA_ileon.csv”	10.15454/QSTXWF
16S rDNA sequences	NCBI SRA	PRJNA615661
OTU table and taxonomic affiliations	Data INRAE, “caecal_appendix_microbiome_rabbit_newSILVAdatabase.rdata” and “OTU_abundances.xlsx”	10.15454/QSTXWF
Unstratified pathway abundance per sample (from PICRUST2)	Data INRAE, “Path_abun_unstrat_transpo.csv”	10.15454/QSTXWF
Microbiota analysis	Data INRAE, “Within_distance_wunifrac.csv”	10.15454/QSTXWF
Metabolites quantification	Data INRAE, “Metabolites_caecum.csv”	10.15454/QSTXWF
Nest quality assessment using infrared camera	DATA INRAE, Files from “J1_1802_101” to “J1_1802_329”	10.15454/QSTXWF

Raw 16S rRNA gene reads files (fastq format) were deposited in the National Center for Biotechnology Information Sequence Read Archive (NCBI accession PRJNA615661^18^, see Table 2). All other raw and processed data used for analysis are provided in Data INRAE repository^19^ [10.15454/QSTXWF].

**Table 2:** Metadata for 16S rRNA gene amplicons sequencing.

accession	study	bioproject_accession	biosample_accession	sample_ID	luminal_content	library_ID	title	library_strategy	library_source	library_selection	library_layout	platform	instrument_model	design_description	filetype	filename	filename2
SRR11430062	SRP254184	PRJNA615661	SAMN14464043	301	caecum	CP1802-cc301_TCCATT	16S rDNA caecum	AMPLICON	METAGENOMIC	PCR	paired	ILLUMINA	Illumina MiSeq	PCR of V3-V4 DNA16S region	fastq	CP1802-cc301_TCCATT-C43D4_L001_R1.fastq.gz	CP1802-cc301_TCCATT-C43D4_L001_R2.fastq.gz
SRR11430061	SRP254184	PRJNA615661	SAMN14464044	302	caecum	CP1802-cc302_AAACAA	16S rDNA caecum	AMPLICON	METAGENOMIC	PCR	paired	ILLUMINA	Illumina MiSeq	PCR of V3-V4 DNA16S region	fastq	CP1802-cc302_AAACAA-C43D4_L001_R1.fastq.gz	CP1802-cc302_AAACAA-C43D4_L001_R2.fastq.gz
SRR11429850	SRP254184	PRJNA615661	SAMN14464045	303	caecum	CP1802-cc303_AATAAT	16S rDNA caecum	AMPLICON	METAGENOMIC	PCR	paired	ILLUMINA	Illumina MiSeq	PCR of V3-V4 DNA16S region	fastq	CP1802-cc303_AATAAT-C43D4_L001_R1.fastq.gz	CP1802-cc303_AATAAT-C43D4_L001_R2.fastq.gz
SRR11429807	SRP254184	PRJNA615661	SAMN14464046	304	caecum	CP1802-cc304_GCCCGA	16S rDNA caecum	AMPLICON	METAGENOMIC	PCR	paired	ILLUMINA	Illumina MiSeq	PCR of V3-V4 DNA16S region	fastq	CP1802-cc304_GCCCGA-C43D4_L001_R1.fastq.gz	CP1802-cc304_GCCCGA-C43D4_L001_R2.fastq.gz
SRR11429928	SRP254184	PRJNA615661	SAMN14464047	307	caecum	CP1802-cc307_ACCGAA	16S rDNA caecum	AMPLICON	METAGENOMIC	PCR	paired	ILLUMINA	Illumina MiSeq	PCR of V3-V4 DNA16S region	fastq	CP1802-cc307_ACCGAA-C43D4_L001_R1.fastq.gz	CP1802-cc307_ACCGAA-C43D4_L001_R2.fastq.gz
SRR11429917	SRP254184	PRJNA615661	SAMN14464048	309	caecum	CP1802-cc309_ACCTCA	16S rDNA caecum	AMPLICON	METAGENOMIC	PCR	paired	ILLUMINA	Illumina MiSeq	PCR of V3-V4 DNA16S region	fastq	CP1802-cc309_ACCTCA-C43D4_L001_R1.fastq.gz	CP1802-cc309_ACCTCA-C43D4_L001_R2.fastq.gz
SRR11429906	SRP254184	PRJNA615661	SAMN14464049	310	caecum	CP1802-cc310_AGGGAT	16S rDNA caecum	AMPLICON	METAGENOMIC	PCR	paired	ILLUMINA	Illumina MiSeq	PCR of V3-V4 DNA16S region	fastq	CP1802-cc310_AGGGAT-C43D4_L001_R1.fastq.gz	CP1802-cc310_AGGGAT-C43D4_L001_R2.fastq.gz
SRR11430091	SRP254184	PRJNA615661	SAMN14464050	312	caecum	CP1802-cc312_TGTCTA	16S rDNA caecum	AMPLICON	METAGENOMIC	PCR	paired	ILLUMINA	Illumina MiSeq	PCR of V3-V4 DNA16S region	fastq	CP1802-cc312_TGTCTA-C43D4_L001_R1.fastq.gz	CP1802-cc312_TGTCTA-C43D4_L001_R2.fastq.gz
SRR11430080	SRP254184	PRJNA615661	SAMN14464051	313	caecum	CP1802-cc313_CGCATT	16S rDNA caecum	AMPLICON	METAGENOMIC	PCR	paired	ILLUMINA	Illumina MiSeq	PCR of V3-V4 DNA16S region	fastq	CP1802-cc313_CGCATT-C43D4_L001_R1.fastq.gz	CP1802-cc313_CGCATT-C43D4_L001_R2.fastq.gz
SRR11430069	SRP254184	PRJNA615661	SAMN14464052	314	caecum	CP1802-cc314_CCCACG	16S rDNA caecum	AMPLICON	METAGENOMIC	PCR	paired	ILLUMINA	Illumina MiSeq	PCR of V3-V4 DNA16S region	fastq	CP1802-cc314_CCCACG-C43D4_L001_R1.fastq.gz	CP1802-cc314_CCCACG-C43D4_L001_R2.fastq.gz
SRR11430060	SRP254184	PRJNA615661	SAMN14464053	317	caecum	CP1802-cc317_CCCGAG	16S rDNA caecum	AMPLICON	METAGENOMIC	PCR	paired	ILLUMINA	Illumina MiSeq	PCR of V3-V4 DNA16S region	fastq	CP1802-cc317_CCCGAG-C43D4_L001_R1.fastq.gz	CP1802-cc317_CCCGAG-C43D4_L001_R2.fastq.gz
SRR11430049	SRP254184	PRJNA615661	SAMN14464054	319	caecum	CP1802-cc319_TTCCGT	16S rDNA caecum	AMPLICON	METAGENOMIC	PCR	paired	ILLUMINA	Illumina MiSeq	PCR of V3-V4 DNA16S region	fastq	CP1802-cc319_TTCCGT-C43D4_L001_R1.fastq.gz	CP1802-cc319_TTCCGT-C43D4_L001_R2.fastq.gz
SRR11430038	SRP254184	PRJNA615661	SAMN14464055	320	caecum	CP1802-cc320_ATATCC	16S rDNA caecum	AMPLICON	METAGENOMIC	PCR	paired	ILLUMINA	Illumina MiSeq	PCR of V3-V4 DNA16S region	fastq	CP1802-cc320_ATATCC-C43D4_L001_R1.fastq.gz	CP1802-cc320_ATATCC-C43D4_L001_R2.fastq.gz
SRR11430027	SRP254184	PRJNA615661	SAMN14464056	321	caecum	CP1802-cc321_TCGGCC	16S rDNA caecum	AMPLICON	METAGENOMIC	PCR	paired	ILLUMINA	Illumina MiSeq	PCR of V3-V4 DNA16S region	fastq	CP1802-cc321_TCGGCC-C43D4_L001_R1.fastq.gz	CP1802-cc321_TCGGCC-C43D4_L001_R2.fastq.gz
SRR11430016	SRP254184	PRJNA615661	SAMN14464057	322	caecum	CP1802-cc322_GTCAGG	16S rDNA caecum	AMPLICON	METAGENOMIC	PCR	paired	ILLUMINA	Illumina MiSeq	PCR of V3-V4 DNA16S region	fastq	CP1802-cc322_GTCAGG-C43D4_L001_R1.fastq.gz	CP1802-cc322_GTCAGG-C43D4_L001_R2.fastq.gz
SRR11430005	SRP254184	PRJNA615661	SAMN14464058	324	caecum	CP1802-cc324_TAACCG	16S rDNA caecum	AMPLICON	METAGENOMIC	PCR	paired	ILLUMINA	Illumina MiSeq	PCR of V3-V4 DNA16S region	fastq	CP1802-cc324_TAACCG-C43D4_L001_R1.fastq.gz	CP1802-cc324_TAACCG-C43D4_L001_R2.fastq.gz
SRR11429894	SRP254184	PRJNA615661	SAMN14464059	326	caecum	CP1802-cc326_CAATTA	16S rDNA caecum	AMPLICON	METAGENOMIC	PCR	paired	ILLUMINA	Illumina MiSeq	PCR of V3-V4 DNA16S region	fastq	CP1802-cc326_CAATTA-C43D4_L001_R1.fastq.gz	CP1802-cc326_CAATTA-C43D4_L001_R2.fastq.gz
SRR11429883	SRP254184	PRJNA615661	SAMN14464060	327	caecum	CP1802-cc327_AGTGTC	16S rDNA caecum	AMPLICON	METAGENOMIC	PCR	paired	ILLUMINA	Illumina MiSeq	PCR of V3-V4 DNA16S region	fastq	CP1802-cc327_AGTGTC-C43D4_L001_R1.fastq.gz	CP1802-cc327_AGTGTC-C43D4_L001_R2.fastq.gz
SRR11429872	SRP254184	PRJNA615661	SAMN14464061	328	caecum	CP1802-cc328_ATTGAG	16S rDNA caecum	AMPLICON	METAGENOMIC	PCR	paired	ILLUMINA	Illumina MiSeq	PCR of V3-V4 DNA16S region	fastq	CP1802-cc328_ATTGAG-C43D4_L001_R1.fastq.gz	CP1802-cc328_ATTGAG-C43D4_L001_R2.fastq.gz
SRR11429861	SRP254184	PRJNA615661	SAMN14464062	329	caecum	CP1802-cc329_TATTGG	16S rDNA caecum	AMPLICON	METAGENOMIC	PCR	paired	ILLUMINA	Illumina MiSeq	PCR of V3-V4 DNA16S region	fastq	CP1802-cc329_TATTGG-C43D4_L001_R1.fastq.gz	CP1802-cc329_TATTGG-C43D4_L001_R2.fastq.gz
SRR11429849	SRP254184	PRJNA615661	SAMN14464063	330	caecum	CP1802-cc330_AACGCA	16S rDNA caecum	AMPLICON	METAGENOMIC	PCR	paired	ILLUMINA	Illumina MiSeq	PCR of V3-V4 DNA16S region	fastq	CP1802-cc330_AACGCA-C43D4_L001_R1.fastq.gz	CP1802-cc330_AACGCA-C43D4_L001_R2.fastq.gz
SRR11429838	SRP254184	PRJNA615661	SAMN14464064	331	caecum	CP1802-cc331_ACGTCG	16S rDNA caecum	AMPLICON	METAGENOMIC	PCR	paired	ILLUMINA	Illumina MiSeq	PCR of V3-V4 DNA16S region	fastq	CP1802-cc331_ACGTCG-C43D4_L001_R1.fastq.gz	CP1802-cc331_ACGTCG-C43D4_L001_R2.fastq.gz
SRR11429991	SRP254184	PRJNA615661	SAMN14464065	332	caecum	CP1802-cc332_GTACAG	16S rDNA caecum	AMPLICON	METAGENOMIC	PCR	paired	ILLUMINA	Illumina MiSeq	PCR of V3-V4 DNA16S region	fastq	CP1802-cc332_GTACAG-C43D4_L001_R1.fastq.gz	CP1802-cc332_GTACAG-C43D4_L001_R2.fastq.gz
SRR11429980	SRP254184	PRJNA615661	SAMN14464066	333	caecum	CP1802-cc333_GTGCTG	16S rDNA caecum	AMPLICON	METAGENOMIC	PCR	paired	ILLUMINA	Illumina MiSeq	PCR of V3-V4 DNA16S region	fastq	CP1802-cc333_GTGCTG-C43D4_L001_R1.fastq.gz	CP1802-cc333_GTGCTG-C43D4_L001_R2.fastq.gz
SRR11429969	SRP254184	PRJNA615661	SAMN14464067	334	caecum	CP1802-cc334_CAATGC	16S rDNA caecum	AMPLICON	METAGENOMIC	PCR	paired	ILLUMINA	Illumina MiSeq	PCR of V3-V4 DNA16S region	fastq	CP1802-cc334_CAATGC-C43D4_L001_R1.fastq.gz	CP1802-cc334_CAATGC-C43D4_L001_R2.fastq.gz
SRR11429958	SRP254184	PRJNA615661	SAMN14464068	335	caecum	CP1802-cc335_CCGTAG	16S rDNA caecum	AMPLICON	METAGENOMIC	PCR	paired	ILLUMINA	Illumina MiSeq	PCR of V3-V4 DNA16S region	fastq	CP1802-cc335_CCGTAG-C43D4_L001_R1.fastq.gz	CP1802-cc335_CCGTAG-C43D4_L001_R2.fastq.gz
SRR11429947	SRP254184	PRJNA615661	SAMN14464069	337	caecum	CP1802-cc337_CTCCCC	16S rDNA caecum	AMPLICON	METAGENOMIC	PCR	paired	ILLUMINA	Illumina MiSeq	PCR of V3-V4 DNA16S region	fastq	CP1802-cc337_CTCCCC-C43D4_L001_R1.fastq.gz	CP1802-cc337_CTCCCC-C43D4_L001_R2.fastq.gz
SRR11429936	SRP254184	PRJNA615661	SAMN14464070	338	caecum	CP1802-cc338_GACTAC	16S rDNA caecum	AMPLICON	METAGENOMIC	PCR	paired	ILLUMINA	Illumina MiSeq	PCR of V3-V4 DNA16S region	fastq	CP1802-cc338_GACTAC-C43D4_L001_R1.fastq.gz	CP1802-cc338_GACTAC-C43D4_L001_R2.fastq.gz
SRR11429825	SRP254184	PRJNA615661	SAMN14464071	340	caecum	CP1802-cc340_CATAAC	16S rDNA caecum	AMPLICON	METAGENOMIC	PCR	paired	ILLUMINA	Illumina MiSeq	PCR of V3-V4 DNA16S region	fastq	CP1802-cc340_CATAAC-C43D4_L001_R1.fastq.gz	CP1802-cc340_CATAAC-C43D4_L001_R2.fastq.gz
SRR11429814	SRP254184	PRJNA615661	SAMN14464072	341	caecum	CP1802-cc341_CCCTCC	16S rDNA caecum	AMPLICON	METAGENOMIC	PCR	paired	ILLUMINA	Illumina MiSeq	PCR of V3-V4 DNA16S region	fastq	CP1802-cc341_CCCTCC-C43D4_L001_R1.fastq.gz	CP1802-cc341_CCCTCC-C43D4_L001_R2.fastq.gz
SRR11429806	SRP254184	PRJNA615661	SAMN14464073	342	caecum	CP1802-cc342_TGTTCG	16S rDNA caecum	AMPLICON	METAGENOMIC	PCR	paired	ILLUMINA	Illumina MiSeq	PCR of V3-V4 DNA16S region	fastq	CP1802-cc342_TGTTCG-C43D4_L001_R1.fastq.gz	CP1802-cc342_TGTTCG-C43D4_L001_R2.fastq.gz
SRR11429805	SRP254184	PRJNA615661	SAMN14464074	343	caecum	CP1802-cc343_CATGAG	16S rDNA caecum	AMPLICON	METAGENOMIC	PCR	paired	ILLUMINA	Illumina MiSeq	PCR of V3-V4 DNA16S region	fastq	CP1802-cc343_CATGAG-C43D4_L001_R1.fastq.gz	CP1802-cc343_CATGAG-C43D4_L001_R2.fastq.gz
SRR11429804	SRP254184	PRJNA615661	SAMN14464075	344	caecum	CP1802-cc344_AAGTGG	16S rDNA caecum	AMPLICON	METAGENOMIC	PCR	paired	ILLUMINA	Illumina MiSeq	PCR of V3-V4 DNA16S region	fastq	CP1802-cc344_AAGTGG-C43D4_L001_R1.fastq.gz	CP1802-cc344_AAGTGG-C43D4_L001_R2.fastq.gz
SRR11429803	SRP254184	PRJNA615661	SAMN14464076	347	caecum	CP1802-cc347_CTTTAG	16S rDNA caecum	AMPLICON	METAGENOMIC	PCR	paired	ILLUMINA	Illumina MiSeq	PCR of V3-V4 DNA16S region	fastq	CP1802-cc347_CTTTAG-C43D4_L001_R1.fastq.gz	CP1802-cc347_CTTTAG-C43D4_L001_R2.fastq.gz
SRR11429802	SRP254184	PRJNA615661	SAMN14464077	349	caecum	CP1802-cc349_GAGAAC	16S rDNA caecum	AMPLICON	METAGENOMIC	PCR	paired	ILLUMINA	Illumina MiSeq	PCR of V3-V4 DNA16S region	fastq	CP1802-cc349_GAGAAC-C43D4_L001_R1.fastq.gz	CP1802-cc349_GAGAAC-C43D4_L001_R2.fastq.gz
SRR11429801	SRP254184	PRJNA615661	SAMN14464078	350	caecum	CP1802-cc350_CCAACA	16S rDNA caecum	AMPLICON	METAGENOMIC	PCR	paired	ILLUMINA	Illumina MiSeq	PCR of V3-V4 DNA16S region	fastq	CP1802-cc350_CCAACA-C43D4_L001_R1.fastq.gz	CP1802-cc350_CCAACA-C43D4_L001_R2.fastq.gz
SRR11429800	SRP254184	PRJNA615661	SAMN14464079	352	caecum	CP1802-cc352_GTCAAA	16S rDNA caecum	AMPLICON	METAGENOMIC	PCR	paired	ILLUMINA	Illumina MiSeq	PCR of V3-V4 DNA16S region	fastq	CP1802-cc352_GTCAAA-C43D4_L001_R1.fastq.gz	CP1802-cc352_GTCAAA-C43D4_L001_R2.fastq.gz
SRR11429799	SRP254184	PRJNA615661	SAMN14464080	353	caecum	CP1802-cc353_CCCTGG	16S rDNA caecum	AMPLICON	METAGENOMIC	PCR	paired	ILLUMINA	Illumina MiSeq	PCR of V3-V4 DNA16S region	fastq	CP1802-cc353_CCCTGG-C43D4_L001_R1.fastq.gz	CP1802-cc353_CCCTGG-C43D4_L001_R2.fastq.gz
SRR11429930	SRP254184	PRJNA615661	SAMN14464081	354	caecum	CP1802-cc354_CGCCAC	16S rDNA caecum	AMPLICON	METAGENOMIC	PCR	paired	ILLUMINA	Illumina MiSeq	PCR of V3-V4 DNA16S region	fastq	CP1802-cc354_CGCCAC-C43D4_L001_R1.fastq.gz	CP1802-cc354_CGCCAC-C43D4_L001_R2.fastq.gz
SRR11429929	SRP254184	PRJNA615661	SAMN14464082	356	caecum	CP1802-cc356_TAATAT	16S rDNA caecum	AMPLICON	METAGENOMIC	PCR	paired	ILLUMINA	Illumina MiSeq	PCR of V3-V4 DNA16S region	fastq	CP1802-cc356_TAATAT-C43D4_L001_R1.fastq.gz	CP1802-cc356_TAATAT-C43D4_L001_R2.fastq.gz
SRR11429927	SRP254184	PRJNA615661	SAMN14464083	357	caecum	CP1802-cc357_TGGTGT	16S rDNA caecum	AMPLICON	METAGENOMIC	PCR	paired	ILLUMINA	Illumina MiSeq	PCR of V3-V4 DNA16S region	fastq	CP1802-cc357_TGGTGT-C43D4_L001_R1.fastq.gz	CP1802-cc357_TGGTGT-C43D4_L001_R2.fastq.gz
SRR11429926	SRP254184	PRJNA615661	SAMN14464084	358	caecum	CP1802-cc358_CTACCG	16S rDNA caecum	AMPLICON	METAGENOMIC	PCR	paired	ILLUMINA	Illumina MiSeq	PCR of V3-V4 DNA16S region	fastq	CP1802-cc358_CTACCG-C43D4_L001_R1.fastq.gz	CP1802-cc358_CTACCG-C43D4_L001_R2.fastq.gz
SRR11429925	SRP254184	PRJNA615661	SAMN14464085	359	caecum	CP1802-cc359_CGTGCG	16S rDNA caecum	AMPLICON	METAGENOMIC	PCR	paired	ILLUMINA	Illumina MiSeq	PCR of V3-V4 DNA16S region	fastq	CP1802-cc359_CGTGCG-C43D4_L001_R1.fastq.gz	CP1802-cc359_CGTGCG-C43D4_L001_R2.fastq.gz
SRR11429924	SRP254184	PRJNA615661	SAMN14464086	360	caecum	CP1802-cc360_GATTCA	16S rDNA caecum	AMPLICON	METAGENOMIC	PCR	paired	ILLUMINA	Illumina MiSeq	PCR of V3-V4 DNA16S region	fastq	CP1802-cc360_GATTCA-C43D4_L001_R1.fastq.gz	CP1802-cc360_GATTCA-C43D4_L001_R2.fastq.gz
SRR11429923	SRP254184	PRJNA615661	SAMN14464087	362	caecum	CP1802-cc362_TACCAA	16S rDNA caecum	AMPLICON	METAGENOMIC	PCR	paired	ILLUMINA	Illumina MiSeq	PCR of V3-V4 DNA16S region	fastq	CP1802-cc362_TACCAA-C43D4_L001_R1.fastq.gz	CP1802-cc362_TACCAA-C43D4_L001_R2.fastq.gz
SRR11429922	SRP254184	PRJNA615661	SAMN14464088	364	caecum	CP1802-cc364_CGTACT	16S rDNA caecum	AMPLICON	METAGENOMIC	PCR	paired	ILLUMINA	Illumina MiSeq	PCR of V3-V4 DNA16S region	fastq	CP1802-cc364_CGTACT-C43D4_L001_R1.fastq.gz	CP1802-cc364_CGTACT-C43D4_L001_R2.fastq.gz
SRR11429921	SRP254184	PRJNA615661	SAMN14464089	366	caecum	CP1802-cc366_CACACT	16S rDNA caecum	AMPLICON	METAGENOMIC	PCR	paired	ILLUMINA	Illumina MiSeq	PCR of V3-V4 DNA16S region	fastq	CP1802-cc366_CACACT-C43D4_L001_R1.fastq.gz	CP1802-cc366_CACACT-C43D4_L001_R2.fastq.gz
SRR11429920	SRP254184	PRJNA615661	SAMN14464090	367	caecum	CP1802-cc367_GAGCAA	16S rDNA caecum	AMPLICON	METAGENOMIC	PCR	paired	ILLUMINA	Illumina MiSeq	PCR of V3-V4 DNA16S region	fastq	CP1802-cc367_GAGCAA-C9F6J_L001_R1.fastq.gz	CP1802-cc367_GAGCAA-C9F6J_L001_R2.fastq.gz
SRR11429919	SRP254184	PRJNA615661	SAMN14464091	368	caecum	CP1802-cc368_ACTTTT	16S rDNA caecum	AMPLICON	METAGENOMIC	PCR	paired	ILLUMINA	Illumina MiSeq	PCR of V3-V4 DNA16S region	fastq	CP1802-cc368_ACTTTT-C9F6J_L001_R1.fastq.gz	CP1802-cc368_ACTTTT-C9F6J_L001_R2.fastq.gz
SRR11429918	SRP254184	PRJNA615661	SAMN14464092	369	caecum	CP1802-cc369_ACTCGA	16S rDNA caecum	AMPLICON	METAGENOMIC	PCR	paired	ILLUMINA	Illumina MiSeq	PCR of V3-V4 DNA16S region	fastq	CP1802-cc369_ACTCGA-C43D4_L001_R1.fastq.gz	CP1802-cc369_ACTCGA-C43D4_L001_R2.fastq.gz
SRR11429916	SRP254184	PRJNA615661	SAMN14464093	370	caecum	CP1802-cc370_TACTCA	16S rDNA caecum	AMPLICON	METAGENOMIC	PCR	paired	ILLUMINA	Illumina MiSeq	PCR of V3-V4 DNA16S region	fastq	CP1802-cc370_TACTCA-C9F6J_L001_R1.fastq.gz	CP1802-cc370_TACTCA-C9F6J_L001_R2.fastq.gz
SRR11429915	SRP254184	PRJNA615661	SAMN14464094	371	caecum	CP1802-cc371_CAGAGC	16S rDNA caecum	AMPLICON	METAGENOMIC	PCR	paired	ILLUMINA	Illumina MiSeq	PCR of V3-V4 DNA16S region	fastq	CP1802-cc371_CAGAGC-C43D4_L001_R1.fastq.gz	CP1802-cc371_CAGAGC-C43D4_L001_R2.fastq.gz
SRR11429914	SRP254184	PRJNA615661	SAMN14464095	372	caecum	CP1802-cc372_GGCCTC	16S rDNA caecum	AMPLICON	METAGENOMIC	PCR	paired	ILLUMINA	Illumina MiSeq	PCR of V3-V4 DNA16S region	fastq	CP1802-cc372_GGCCTC-C9F6J_L001_R1.fastq.gz	CP1802-cc372_GGCCTC-C9F6J_L001_R2.fastq.gz
SRR11429913	SRP254184	PRJNA615661	SAMN14464096	373	caecum	CP1802-cc373_AATCAC	16S rDNA caecum	AMPLICON	METAGENOMIC	PCR	paired	ILLUMINA	Illumina MiSeq	PCR of V3-V4 DNA16S region	fastq	CP1802-cc373_AATCAC-C43D4_L001_R1.fastq.gz	CP1802-cc373_AATCAC-C43D4_L001_R2.fastq.gz
SRR11429912	SRP254184	PRJNA615661	SAMN14464097	374	caecum	CP1802-cc374_CCCGGC	16S rDNA caecum	AMPLICON	METAGENOMIC	PCR	paired	ILLUMINA	Illumina MiSeq	PCR of V3-V4 DNA16S region	fastq	CP1802-cc374_CCCGGC-C9F6J_L001_R1.fastq.gz	CP1802-cc374_CCCGGC-C9F6J_L001_R2.fastq.gz
SRR11429911	SRP254184	PRJNA615661	SAMN14464098	375	caecum	CP1802-cc375_TCCCCA	16S rDNA caecum	AMPLICON	METAGENOMIC	PCR	paired	ILLUMINA	Illumina MiSeq	PCR of V3-V4 DNA16S region	fastq	CP1802-cc375_TCCCCA-C43D4_L001_R1.fastq.gz	CP1802-cc375_TCCCCA-C43D4_L001_R2.fastq.gz
SRR11429910	SRP254184	PRJNA615661	SAMN14464099	377	caecum	CP1802-cc377_CTCTCG	16S rDNA caecum	AMPLICON	METAGENOMIC	PCR	paired	ILLUMINA	Illumina MiSeq	PCR of V3-V4 DNA16S region	fastq	CP1802-cc377_CTCTCG-C43D4_L001_R1.fastq.gz	CP1802-cc377_CTCTCG-C43D4_L001_R2.fastq.gz
SRR11429909	SRP254184	PRJNA615661	SAMN14464100	378	caecum	CP1802-cc378_CAGATG	16S rDNA caecum	AMPLICON	METAGENOMIC	PCR	paired	ILLUMINA	Illumina MiSeq	PCR of V3-V4 DNA16S region	fastq	CP1802-cc378_CAGATG-C43D4_L001_R1.fastq.gz	CP1802-cc378_CAGATG-C43D4_L001_R2.fastq.gz
SRR11429908	SRP254184	PRJNA615661	SAMN14464101	380	caecum	CP1802-cc380_GCCGGT	16S rDNA caecum	AMPLICON	METAGENOMIC	PCR	paired	ILLUMINA	Illumina MiSeq	PCR of V3-V4 DNA16S region	fastq	CP1802-cc380_GCCGGT-C9F6J_L001_R1.fastq.gz	CP1802-cc380_GCCGGT-C9F6J_L001_R2.fastq.gz
SRR11429907	SRP254184	PRJNA615661	SAMN14464102	381	caecum	CP1802-cc381_CAGTCT	16S rDNA caecum	AMPLICON	METAGENOMIC	PCR	paired	ILLUMINA	Illumina MiSeq	PCR of V3-V4 DNA16S region	fastq	CP1802-cc381_CAGTCT-C43D4_L001_R1.fastq.gz	CP1802-cc381_CAGTCT-C43D4_L001_R2.fastq.gz
SRR11429905	SRP254184	PRJNA615661	SAMN14464103	382	caecum	CP1802-cc382_AAATTG	16S rDNA caecum	AMPLICON	METAGENOMIC	PCR	paired	ILLUMINA	Illumina MiSeq	PCR of V3-V4 DNA16S region	fastq	CP1802-cc382_AAATTG-C43D4_L001_R1.fastq.gz	CP1802-cc382_AAATTG-C43D4_L001_R2.fastq.gz
SRR11429904	SRP254184	PRJNA615661	SAMN14464104	383	caecum	CP1802-cc383_AGTTTG	16S rDNA caecum	AMPLICON	METAGENOMIC	PCR	paired	ILLUMINA	Illumina MiSeq	PCR of V3-V4 DNA16S region	fastq	CP1802-cc383_AGTTTG-C43D4_L001_R1.fastq.gz	CP1802-cc383_AGTTTG-C43D4_L001_R2.fastq.gz
SRR11429903	SRP254184	PRJNA615661	SAMN14464105	384	caecum	CP1802-cc384_TCTCGG	16S rDNA caecum	AMPLICON	METAGENOMIC	PCR	paired	ILLUMINA	Illumina MiSeq	PCR of V3-V4 DNA16S region	fastq	CP1802-cc384_TCTCGG-C43D4_L001_R1.fastq.gz	CP1802-cc384_TCTCGG-C43D4_L001_R2.fastq.gz
SRR11429902	SRP254184	PRJNA615661	SAMN14464106	387	caecum	CP1802-cc387_ACTGCG	16S rDNA caecum	AMPLICON	METAGENOMIC	PCR	paired	ILLUMINA	Illumina MiSeq	PCR of V3-V4 DNA16S region	fastq	CP1802-cc387_ACTGCG-C9F6J_L001_R1.fastq.gz	CP1802-cc387_ACTGCG-C9F6J_L001_R2.fastq.gz
SRR11429901	SRP254184	PRJNA615661	SAMN14464107	389	caecum	CP1802-cc389_ATTAGG	16S rDNA caecum	AMPLICON	METAGENOMIC	PCR	paired	ILLUMINA	Illumina MiSeq	PCR of V3-V4 DNA16S region	fastq	CP1802-cc389_ATTAGG-C9F6J_L001_R1.fastq.gz	CP1802-cc389_ATTAGG-C9F6J_L001_R2.fastq.gz
SRR11429900	SRP254184	PRJNA615661	SAMN14464108	390	caecum	CP1802-cc390_ACAGTT	16S rDNA caecum	AMPLICON	METAGENOMIC	PCR	paired	ILLUMINA	Illumina MiSeq	PCR of V3-V4 DNA16S region	fastq	CP1802-cc390_ACAGTT-C43D4_L001_R1.fastq.gz	CP1802-cc390_ACAGTT-C43D4_L001_R2.fastq.gz
SRR11429899	SRP254184	PRJNA615661	SAMN14464109	392	caecum	CP1802-cc392_GCTATC	16S rDNA caecum	AMPLICON	METAGENOMIC	PCR	paired	ILLUMINA	Illumina MiSeq	PCR of V3-V4 DNA16S region	fastq	CP1802-cc392_GCTATC-C43D4_L001_R1.fastq.gz	CP1802-cc392_GCTATC-C43D4_L001_R2.fastq.gz
SRR11430094	SRP254184	PRJNA615661	SAMN14464110	393	caecum	CP1802-cc393_TTAAAT	16S rDNA caecum	AMPLICON	METAGENOMIC	PCR	paired	ILLUMINA	Illumina MiSeq	PCR of V3-V4 DNA16S region	fastq	CP1802-cc393_TTAAAT-C43D4_L001_R1.fastq.gz	CP1802-cc393_TTAAAT-C43D4_L001_R2.fastq.gz
SRR11430093	SRP254184	PRJNA615661	SAMN14464111	394	caecum	CP1802-cc394_TTTGTA	16S rDNA caecum	AMPLICON	METAGENOMIC	PCR	paired	ILLUMINA	Illumina MiSeq	PCR of V3-V4 DNA16S region	fastq	CP1802-cc394_TTTGTA-C43D4_L001_R1.fastq.gz	CP1802-cc394_TTTGTA-C43D4_L001_R2.fastq.gz
SRR11430092	SRP254184	PRJNA615661	SAMN14464112	396	caecum	CP1802-cc396_GTTACC	16S rDNA caecum	AMPLICON	METAGENOMIC	PCR	paired	ILLUMINA	Illumina MiSeq	PCR of V3-V4 DNA16S region	fastq	CP1802-cc396_GTTACC-C43D4_L001_R1.fastq.gz	CP1802-cc396_GTTACC-C43D4_L001_R2.fastq.gz
SRR11430090	SRP254184	PRJNA615661	SAMN14464113	397	caecum	CP1802-cc397_CCATTG	16S rDNA caecum	AMPLICON	METAGENOMIC	PCR	paired	ILLUMINA	Illumina MiSeq	PCR of V3-V4 DNA16S region	fastq	CP1802-cc397_CCATTG-C43D4_L001_R1.fastq.gz	CP1802-cc397_CCATTG-C43D4_L001_R2.fastq.gz
SRR11430089	SRP254184	PRJNA615661	SAMN14464114	398	caecum	CP1802-cc398_TCGCGC	16S rDNA caecum	AMPLICON	METAGENOMIC	PCR	paired	ILLUMINA	Illumina MiSeq	PCR of V3-V4 DNA16S region	fastq	CP1802-cc398_TCGCGC-C43D4_L001_R1.fastq.gz	CP1802-cc398_TCGCGC-C43D4_L001_R2.fastq.gz
SRR11430088	SRP254184	PRJNA615661	SAMN14464115	399	caecum	CP1802-cc399_TAACTT	16S rDNA caecum	AMPLICON	METAGENOMIC	PCR	paired	ILLUMINA	Illumina MiSeq	PCR of V3-V4 DNA16S region	fastq	CP1802-cc399_TAACTT-C43D4_L001_R1.fastq.gz	CP1802-cc399_TAACTT-C43D4_L001_R2.fastq.gz
SRR11430087	SRP254184	PRJNA615661	SAMN14464116	400	caecum	CP1802-cc400_GGACTT	16S rDNA caecum	AMPLICON	METAGENOMIC	PCR	paired	ILLUMINA	Illumina MiSeq	PCR of V3-V4 DNA16S region	fastq	CP1802-cc400_GGACTT-C9F6J_L001_R1.fastq.gz	CP1802-cc400_GGACTT-C9F6J_L001_R2.fastq.gz
SRR11430086	SRP254184	PRJNA615661	SAMN14464117	402	caecum	CP1802-cc402_TCGAAC	16S rDNA caecum	AMPLICON	METAGENOMIC	PCR	paired	ILLUMINA	Illumina MiSeq	PCR of V3-V4 DNA16S region	fastq	CP1802-cc402_TCGAAC-C9F6J_L001_R1.fastq.gz	CP1802-cc402_TCGAAC-C9F6J_L001_R2.fastq.gz
SRR11430085	SRP254184	PRJNA615661	SAMN14464118	404	caecum	CP1802-cc404_CCGACC	16S rDNA caecum	AMPLICON	METAGENOMIC	PCR	paired	ILLUMINA	Illumina MiSeq	PCR of V3-V4 DNA16S region	fastq	CP1802-cc404_CCGACC-C43D4_L001_R1.fastq.gz	CP1802-cc404_CCGACC-C43D4_L001_R2.fastq.gz
SRR11430084	SRP254184	PRJNA615661	SAMN14464119	406	caecum	CP1802-cc406_CAGTCT	16S rDNA caecum	AMPLICON	METAGENOMIC	PCR	paired	ILLUMINA	Illumina MiSeq	PCR of V3-V4 DNA16S region	fastq	CP1802-cc406_CAGTCT-C9F6J_L001_R1.fastq.gz	CP1802-cc406_CAGTCT-C9F6J_L001_R2.fastq.gz
SRR11430083	SRP254184	PRJNA615661	SAMN14464120	407	caecum	CP1802-cc407_GACAGT	16S rDNA caecum	AMPLICON	METAGENOMIC	PCR	paired	ILLUMINA	Illumina MiSeq	PCR of V3-V4 DNA16S region	fastq	CP1802-cc407_GACAGT-C9F6J_L001_R1.fastq.gz	CP1802-cc407_GACAGT-C9F6J_L001_R2.fastq.gz
SRR11430082	SRP254184	PRJNA615661	SAMN14464121	408	caecum	CP1802-cc408_TTTTTC	16S rDNA caecum	AMPLICON	METAGENOMIC	PCR	paired	ILLUMINA	Illumina MiSeq	PCR of V3-V4 DNA16S region	fastq	CP1802-cc408_TTTTTC-C9F6J_L001_R1.fastq.gz	CP1802-cc408_TTTTTC-C9F6J_L001_R2.fastq.gz
SRR11430081	SRP254184	PRJNA615661	SAMN14464122	409	caecum	CP1802-cc409_TGCCTT	16S rDNA caecum	AMPLICON	METAGENOMIC	PCR	paired	ILLUMINA	Illumina MiSeq	PCR of V3-V4 DNA16S region	fastq	CP1802-cc409_TGCCTT-C9F6J_L001_R1.fastq.gz	CP1802-cc409_TGCCTT-C9F6J_L001_R2.fastq.gz
SRR11430079	SRP254184	PRJNA615661	SAMN14464123	410	caecum	CP1802-cc410_GGTAGC	16S rDNA caecum	AMPLICON	METAGENOMIC	PCR	paired	ILLUMINA	Illumina MiSeq	PCR of V3-V4 DNA16S region	fastq	CP1802-cc410_GGTAGC-C43D4_L001_R1.fastq.gz	CP1802-cc410_GGTAGC-C43D4_L001_R2.fastq.gz
SRR11430078	SRP254184	PRJNA615661	SAMN14464124	411	caecum	CP1802-cc411_GCTATC	16S rDNA caecum	AMPLICON	METAGENOMIC	PCR	paired	ILLUMINA	Illumina MiSeq	PCR of V3-V4 DNA16S region	fastq	CP1802-cc411_GCTATC-C9F6J_L001_R1.fastq.gz	CP1802-cc411_GCTATC-C9F6J_L001_R2.fastq.gz
SRR11430077	SRP254184	PRJNA615661	SAMN14464125	412	caecum	CP1802-cc412_TTAGCT	16S rDNA caecum	AMPLICON	METAGENOMIC	PCR	paired	ILLUMINA	Illumina MiSeq	PCR of V3-V4 DNA16S region	fastq	CP1802-cc412_TTAGCT-C9F6J_L001_R1.fastq.gz	CP1802-cc412_TTAGCT-C9F6J_L001_R2.fastq.gz
SRR11430076	SRP254184	PRJNA615661	SAMN14464126	413	caecum	CP1802-cc413_GCAAAT	16S rDNA caecum	AMPLICON	METAGENOMIC	PCR	paired	ILLUMINA	Illumina MiSeq	PCR of V3-V4 DNA16S region	fastq	CP1802-cc413_GCAAAT-C43D4_L001_R1.fastq.gz	CP1802-cc413_GCAAAT-C43D4_L001_R2.fastq.gz
SRR11430075	SRP254184	PRJNA615661	SAMN14464127	414	caecum	CP1802-cc414_TCGCGC	16S rDNA caecum	AMPLICON	METAGENOMIC	PCR	paired	ILLUMINA	Illumina MiSeq	PCR of V3-V4 DNA16S region	fastq	CP1802-cc414_TCGCGC-C9F6J_L001_R1.fastq.gz	CP1802-cc414_TCGCGC-C9F6J_L001_R2.fastq.gz
SRR11430074	SRP254184	PRJNA615661	SAMN14464128	415	caecum	CP1802-cc415_ACATAT	16S rDNA caecum	AMPLICON	METAGENOMIC	PCR	paired	ILLUMINA	Illumina MiSeq	PCR of V3-V4 DNA16S region	fastq	CP1802-cc415_ACATAT-C43D4_L001_R1.fastq.gz	CP1802-cc415_ACATAT-C43D4_L001_R2.fastq.gz
SRR11430073	SRP254184	PRJNA615661	SAMN14464129	417	caecum	CP1802-cc417_GAGCTT	16S rDNA caecum	AMPLICON	METAGENOMIC	PCR	paired	ILLUMINA	Illumina MiSeq	PCR of V3-V4 DNA16S region	fastq	CP1802-cc417_GAGCTT-C43D4_L001_R1.fastq.gz	CP1802-cc417_GAGCTT-C43D4_L001_R2.fastq.gz
SRR11430072	SRP254184	PRJNA615661	SAMN14464130	418	caecum	CP1802-cc418_CTACGC	16S rDNA caecum	AMPLICON	METAGENOMIC	PCR	paired	ILLUMINA	Illumina MiSeq	PCR of V3-V4 DNA16S region	fastq	CP1802-cc418_CTACGC-C9F6J_L001_R1.fastq.gz	CP1802-cc418_CTACGC-C9F6J_L001_R2.fastq.gz
SRR11430071	SRP254184	PRJNA615661	SAMN14464131	420	caecum	CP1802-cc420_GCCAAG	16S rDNA caecum	AMPLICON	METAGENOMIC	PCR	paired	ILLUMINA	Illumina MiSeq	PCR of V3-V4 DNA16S region	fastq	CP1802-cc420_GCCAAG-C43D4_L001_R1.fastq.gz	CP1802-cc420_GCCAAG-C43D4_L001_R2.fastq.gz
SRR11430070	SRP254184	PRJNA615661	SAMN14464132	421	caecum	CP1802-cc421_TGCTGA	16S rDNA caecum	AMPLICON	METAGENOMIC	PCR	paired	ILLUMINA	Illumina MiSeq	PCR of V3-V4 DNA16S region	fastq	CP1802-cc421_TGCTGA-C9F6J_L001_R1.fastq.gz	CP1802-cc421_TGCTGA-C9F6J_L001_R2.fastq.gz
SRR11430068	SRP254184	PRJNA615661	SAMN14464133	422	caecum	CP1802-cc422_TTTTTC	16S rDNA caecum	AMPLICON	METAGENOMIC	PCR	paired	ILLUMINA	Illumina MiSeq	PCR of V3-V4 DNA16S region	fastq	CP1802-cc422_TTTTTC-C43D4_L001_R1.fastq.gz	CP1802-cc422_TTTTTC-C43D4_L001_R2.fastq.gz
SRR11430067	SRP254184	PRJNA615661	SAMN14464134	423	caecum	CP1802-cc423_GTCGTG	16S rDNA caecum	AMPLICON	METAGENOMIC	PCR	paired	ILLUMINA	Illumina MiSeq	PCR of V3-V4 DNA16S region	fastq	CP1802-cc423_GTCGTG-C43D4_L001_R1.fastq.gz	CP1802-cc423_GTCGTG-C43D4_L001_R2.fastq.gz
SRR11430066	SRP254184	PRJNA615661	SAMN14464135	424	caecum	CP1802-cc424_GAGCTT	16S rDNA caecum	AMPLICON	METAGENOMIC	PCR	paired	ILLUMINA	Illumina MiSeq	PCR of V3-V4 DNA16S region	fastq	CP1802-cc424_GAGCTT-C9F6J_L001_R1.fastq.gz	CP1802-cc424_GAGCTT-C9F6J_L001_R2.fastq.gz
SRR11430065	SRP254184	PRJNA615661	SAMN14464136	427	caecum	CP1802-cc427_GCCTAA	16S rDNA caecum	AMPLICON	METAGENOMIC	PCR	paired	ILLUMINA	Illumina MiSeq	PCR of V3-V4 DNA16S region	fastq	CP1802-cc427_GCCTAA-C9F6J_L001_R1.fastq.gz	CP1802-cc427_GCCTAA-C9F6J_L001_R2.fastq.gz
SRR11430064	SRP254184	PRJNA615661	SAMN14464137	429	caecum	CP1802-cc429_TTGCTA	16S rDNA caecum	AMPLICON	METAGENOMIC	PCR	paired	ILLUMINA	Illumina MiSeq	PCR of V3-V4 DNA16S region	fastq	CP1802-cc429_TTGCTA-C9F6J_L001_R1.fastq.gz	CP1802-cc429_TTGCTA-C9F6J_L001_R2.fastq.gz
SRR11430063	SRP254184	PRJNA615661	SAMN14464138	430	caecum	CP1802-cc430_GCAAAT	16S rDNA caecum	AMPLICON	METAGENOMIC	PCR	paired	ILLUMINA	Illumina MiSeq	PCR of V3-V4 DNA16S region	fastq	CP1802-cc430_GCAAAT-C9F6J_L001_R1.fastq.gz	CP1802-cc430_GCAAAT-C9F6J_L001_R2.fastq.gz
SRR11429898	SRP254184	PRJNA615661	SAMN14464139	432	caecum	CP1802-cc432_TACCTG	16S rDNA caecum	AMPLICON	METAGENOMIC	PCR	paired	ILLUMINA	Illumina MiSeq	PCR of V3-V4 DNA16S region	fastq	CP1802-cc432_TACCTG-C9F6J_L001_R1.fastq.gz	CP1802-cc432_TACCTG-C9F6J_L001_R2.fastq.gz
SRR11429897	SRP254184	PRJNA615661	SAMN14464140	433	caecum	CP1802-cc433_GTAACA	16S rDNA caecum	AMPLICON	METAGENOMIC	PCR	paired	ILLUMINA	Illumina MiSeq	PCR of V3-V4 DNA16S region	fastq	CP1802-cc433_GTAACA-C43D4_L001_R1.fastq.gz	CP1802-cc433_GTAACA-C43D4_L001_R2.fastq.gz
SRR11429896	SRP254184	PRJNA615661	SAMN14464141	434	caecum	CP1802-cc434_GTGGGG	16S rDNA caecum	AMPLICON	METAGENOMIC	PCR	paired	ILLUMINA	Illumina MiSeq	PCR of V3-V4 DNA16S region	fastq	CP1802-cc434_GTGGGG-C9F6J_L001_R1.fastq.gz	CP1802-cc434_GTGGGG-C9F6J_L001_R2.fastq.gz
SRR11429895	SRP254184	PRJNA615661	SAMN14464142	436	caecum	CP1802-cc436_TGCTCC	16S rDNA caecum	AMPLICON	METAGENOMIC	PCR	paired	ILLUMINA	Illumina MiSeq	PCR of V3-V4 DNA16S region	fastq	CP1802-cc436_TGCTCC-C9F6J_L001_R1.fastq.gz	CP1802-cc436_TGCTCC-C9F6J_L001_R2.fastq.gz
SRR11430059	SRP254184	PRJNA615661	SAMN14464143	437	caecum	CP1802-cc437_TCACAC	16S rDNA caecum	AMPLICON	METAGENOMIC	PCR	paired	ILLUMINA	Illumina MiSeq	PCR of V3-V4 DNA16S region	fastq	CP1802-cc437_TCACAC-C9F6J_L001_R1.fastq.gz	CP1802-cc437_TCACAC-C9F6J_L001_R2.fastq.gz
SRR11430058	SRP254184	PRJNA615661	SAMN14464144	438	caecum	CP1802-cc438_CTACAT	16S rDNA caecum	AMPLICON	METAGENOMIC	PCR	paired	ILLUMINA	Illumina MiSeq	PCR of V3-V4 DNA16S region	fastq	CP1802-cc438_CTACAT-C9F6J_L001_R1.fastq.gz	CP1802-cc438_CTACAT-C9F6J_L001_R2.fastq.gz
SRR11430057	SRP254184	PRJNA615661	SAMN14464145	439	caecum	CP1802-cc439_GCAGCT	16S rDNA caecum	AMPLICON	METAGENOMIC	PCR	paired	ILLUMINA	Illumina MiSeq	PCR of V3-V4 DNA16S region	fastq	CP1802-cc439_GCAGCT-C43D4_L001_R1.fastq.gz	CP1802-cc439_GCAGCT-C43D4_L001_R2.fastq.gz
SRR11430056	SRP254184	PRJNA615661	SAMN14464146	440	caecum	CP1802-cc440_AACGCA	16S rDNA caecum	AMPLICON	METAGENOMIC	PCR	paired	ILLUMINA	Illumina MiSeq	PCR of V3-V4 DNA16S region	fastq	CP1802-cc440_AACGCA-C9F6J_L001_R1.fastq.gz	CP1802-cc440_AACGCA-C9F6J_L001_R2.fastq.gz
SRR11430055	SRP254184	PRJNA615661	SAMN14464147	442	caecum	CP1802-cc442_GGAGGT	16S rDNA caecum	AMPLICON	METAGENOMIC	PCR	paired	ILLUMINA	Illumina MiSeq	PCR of V3-V4 DNA16S region	fastq	CP1802-cc442_GGAGGT-C43D4_L001_R1.fastq.gz	CP1802-cc442_GGAGGT-C43D4_L001_R2.fastq.gz
SRR11430054	SRP254184	PRJNA615661	SAMN14464148	444	caecum	CP1802-cc444_TGCGGG	16S rDNA caecum	AMPLICON	METAGENOMIC	PCR	paired	ILLUMINA	Illumina MiSeq	PCR of V3-V4 DNA16S region	fastq	CP1802-cc444_TGCGGG-C9F6J_L001_R1.fastq.gz	CP1802-cc444_TGCGGG-C9F6J_L001_R2.fastq.gz
SRR11430053	SRP254184	PRJNA615661	SAMN14464149	446	caecum	CP1802-cc446_GGACGG	16S rDNA caecum	AMPLICON	METAGENOMIC	PCR	paired	ILLUMINA	Illumina MiSeq	PCR of V3-V4 DNA16S region	fastq	CP1802-cc446_GGACGG-C9F6J_L001_R1.fastq.gz	CP1802-cc446_GGACGG-C9F6J_L001_R2.fastq.gz
SRR11430052	SRP254184	PRJNA615661	SAMN14464150	447	caecum	CP1802-cc447_GTTTCT	16S rDNA caecum	AMPLICON	METAGENOMIC	PCR	paired	ILLUMINA	Illumina MiSeq	PCR of V3-V4 DNA16S region	fastq	CP1802-cc447_GTTTCT-C9F6J_L001_R1.fastq.gz	CP1802-cc447_GTTTCT-C9F6J_L001_R2.fastq.gz
SRR11430051	SRP254184	PRJNA615661	SAMN14464151	448	caecum	CP1802-cc448_ATGAAC	16S rDNA caecum	AMPLICON	METAGENOMIC	PCR	paired	ILLUMINA	Illumina MiSeq	PCR of V3-V4 DNA16S region	fastq	CP1802-cc448_ATGAAC-C9F6J_L001_R1.fastq.gz	CP1802-cc448_ATGAAC-C9F6J_L001_R2.fastq.gz
SRR11430050	SRP254184	PRJNA615661	SAMN14464152	449	caecum	CP1802-cc449_TCTATG	16S rDNA caecum	AMPLICON	METAGENOMIC	PCR	paired	ILLUMINA	Illumina MiSeq	PCR of V3-V4 DNA16S region	fastq	CP1802-cc449_TCTATG-C9F6J_L001_R1.fastq.gz	CP1802-cc449_TCTATG-C9F6J_L001_R2.fastq.gz
SRR11430048	SRP254184	PRJNA615661	SAMN14464153	450	caecum	CP1802-cc450_CTAGAG	16S rDNA caecum	AMPLICON	METAGENOMIC	PCR	paired	ILLUMINA	Illumina MiSeq	PCR of V3-V4 DNA16S region	fastq	CP1802-cc450_CTAGAG-C9F6J_L001_R1.fastq.gz	CP1802-cc450_CTAGAG-C9F6J_L001_R2.fastq.gz
SRR11430047	SRP254184	PRJNA615661	SAMN14464154	451	caecum	CP1802-cc451_AATTGC	16S rDNA caecum	AMPLICON	METAGENOMIC	PCR	paired	ILLUMINA	Illumina MiSeq	PCR of V3-V4 DNA16S region	fastq	CP1802-cc451_AATTGC-C9F6J_L001_R1.fastq.gz	CP1802-cc451_AATTGC-C9F6J_L001_R2.fastq.gz
SRR11430046	SRP254184	PRJNA615661	SAMN14464155	452	caecum	CP1802-cc452_ATGCTT	16S rDNA caecum	AMPLICON	METAGENOMIC	PCR	paired	ILLUMINA	Illumina MiSeq	PCR of V3-V4 DNA16S region	fastq	CP1802-cc452_ATGCTT-C9F6J_L001_R1.fastq.gz	CP1802-cc452_ATGCTT-C9F6J_L001_R2.fastq.gz
SRR11430045	SRP254184	PRJNA615661	SAMN14464156	453	caecum	CP1802-cc453_AGAGGG	16S rDNA caecum	AMPLICON	METAGENOMIC	PCR	paired	ILLUMINA	Illumina MiSeq	PCR of V3-V4 DNA16S region	fastq	CP1802-cc453_AGAGGG-C9F6J_L001_R1.fastq.gz	CP1802-cc453_AGAGGG-C9F6J_L001_R2.fastq.gz
SRR11430044	SRP254184	PRJNA615661	SAMN14464157	454	caecum	CP1802-cc454_CCCAAA	16S rDNA caecum	AMPLICON	METAGENOMIC	PCR	paired	ILLUMINA	Illumina MiSeq	PCR of V3-V4 DNA16S region	fastq	CP1802-cc454_CCCAAA-C9F6J_L001_R1.fastq.gz	CP1802-cc454_CCCAAA-C9F6J_L001_R2.fastq.gz
SRR11430043	SRP254184	PRJNA615661	SAMN14464158	455	caecum	CP1802-cc455_GATGCT	16S rDNA caecum	AMPLICON	METAGENOMIC	PCR	paired	ILLUMINA	Illumina MiSeq	PCR of V3-V4 DNA16S region	fastq	CP1802-cc455_GATGCT-C9F6J_L001_R1.fastq.gz	CP1802-cc455_GATGCT-C9F6J_L001_R2.fastq.gz
SRR11430042	SRP254184	PRJNA615661	SAMN14464159	457	caecum	CP1802-cc457_CTCTAC	16S rDNA caecum	AMPLICON	METAGENOMIC	PCR	paired	ILLUMINA	Illumina MiSeq	PCR of V3-V4 DNA16S region	fastq	CP1802-cc457_CTCTAC-C9F6J_L001_R1.fastq.gz	CP1802-cc457_CTCTAC-C9F6J_L001_R2.fastq.gz
SRR11430041	SRP254184	PRJNA615661	SAMN14464160	458	caecum	CP1802-cc458_CAGGAC	16S rDNA caecum	AMPLICON	METAGENOMIC	PCR	paired	ILLUMINA	Illumina MiSeq	PCR of V3-V4 DNA16S region	fastq	CP1802-cc458_CAGGAC-C43D4_L001_R1.fastq.gz	CP1802-cc458_CAGGAC-C43D4_L001_R2.fastq.gz
SRR11430040	SRP254184	PRJNA615661	SAMN14464161	460	caecum	CP1802-cc460_CCTTGA	16S rDNA caecum	AMPLICON	METAGENOMIC	PCR	paired	ILLUMINA	Illumina MiSeq	PCR of V3-V4 DNA16S region	fastq	CP1802-cc460_CCTTGA-C9F6J_L001_R1.fastq.gz	CP1802-cc460_CCTTGA-C9F6J_L001_R2.fastq.gz
SRR11430039	SRP254184	PRJNA615661	SAMN14464162	461	caecum	CP1802-cc461_GTAGAA	16S rDNA caecum	AMPLICON	METAGENOMIC	PCR	paired	ILLUMINA	Illumina MiSeq	PCR of V3-V4 DNA16S region	fastq	CP1802-cc461_GTAGAA-C9F6J_L001_R1.fastq.gz	CP1802-cc461_GTAGAA-C9F6J_L001_R2.fastq.gz
SRR11430037	SRP254184	PRJNA615661	SAMN14464163	463	caecum	CP1802-cc463_CAACAG	16S rDNA caecum	AMPLICON	METAGENOMIC	PCR	paired	ILLUMINA	Illumina MiSeq	PCR of V3-V4 DNA16S region	fastq	CP1802-cc463_CAACAG-C9F6J_L001_R1.fastq.gz	CP1802-cc463_CAACAG-C9F6J_L001_R2.fastq.gz
SRR11430036	SRP254184	PRJNA615661	SAMN14464164	465	caecum	CP1802-cc465_CTTGCA	16S rDNA caecum	AMPLICON	METAGENOMIC	PCR	paired	ILLUMINA	Illumina MiSeq	PCR of V3-V4 DNA16S region	fastq	CP1802-cc465_CTTGCA-C9F6J_L001_R1.fastq.gz	CP1802-cc465_CTTGCA-C9F6J_L001_R2.fastq.gz
SRR11430035	SRP254184	PRJNA615661	SAMN14464165	466	caecum	CP1802-cc466_GGTAGC	16S rDNA caecum	AMPLICON	METAGENOMIC	PCR	paired	ILLUMINA	Illumina MiSeq	PCR of V3-V4 DNA16S region	fastq	CP1802-cc466_GGTAGC-C9F6J_L001_R1.fastq.gz	CP1802-cc466_GGTAGC-C9F6J_L001_R2.fastq.gz
SRR11430034	SRP254184	PRJNA615661	SAMN14464166	467	caecum	CP1802-cc467_TGGATT	16S rDNA caecum	AMPLICON	METAGENOMIC	PCR	paired	ILLUMINA	Illumina MiSeq	PCR of V3-V4 DNA16S region	fastq	CP1802-cc467_TGGATT-C9F6J_L001_R1.fastq.gz	CP1802-cc467_TGGATT-C9F6J_L001_R2.fastq.gz
SRR11430033	SRP254184	PRJNA615661	SAMN14464167	468	caecum	CP1802-cc468_AGGATA	16S rDNA caecum	AMPLICON	METAGENOMIC	PCR	paired	ILLUMINA	Illumina MiSeq	PCR of V3-V4 DNA16S region	fastq	CP1802-cc468_AGGATA-C43D4_L001_R1.fastq.gz	CP1802-cc468_AGGATA-C43D4_L001_R2.fastq.gz
SRR11430032	SRP254184	PRJNA615661	SAMN14464168	469	caecum	CP1802-cc469_TTCGAG	16S rDNA caecum	AMPLICON	METAGENOMIC	PCR	paired	ILLUMINA	Illumina MiSeq	PCR of V3-V4 DNA16S region	fastq	CP1802-cc469_TTCGAG-C9F6J_L001_R1.fastq.gz	CP1802-cc469_TTCGAG-C9F6J_L001_R2.fastq.gz
SRR11430031	SRP254184	PRJNA615661	SAMN14464169	471	caecum	CP1802-cc471_CTGTAA	16S rDNA caecum	AMPLICON	METAGENOMIC	PCR	paired	ILLUMINA	Illumina MiSeq	PCR of V3-V4 DNA16S region	fastq	CP1802-cc471_CTGTAA-C9F6J_L001_R1.fastq.gz	CP1802-cc471_CTGTAA-C9F6J_L001_R2.fastq.gz
SRR11430030	SRP254184	PRJNA615661	SAMN14464170	472	caecum	CP1802-cc472_TCCCCA	16S rDNA caecum	AMPLICON	METAGENOMIC	PCR	paired	ILLUMINA	Illumina MiSeq	PCR of V3-V4 DNA16S region	fastq	CP1802-cc472_TCCCCA-C9F6J_L001_R1.fastq.gz	CP1802-cc472_TCCCCA-C9F6J_L001_R2.fastq.gz
SRR11430029	SRP254184	PRJNA615661	SAMN14464171	474	caecum	CP1802-cc474_GTTCGC	16S rDNA caecum	AMPLICON	METAGENOMIC	PCR	paired	ILLUMINA	Illumina MiSeq	PCR of V3-V4 DNA16S region	fastq	CP1802-cc474_GTTCGC-C9F6J_L001_R1.fastq.gz	CP1802-cc474_GTTCGC-C9F6J_L001_R2.fastq.gz
SRR11430028	SRP254184	PRJNA615661	SAMN14464172	475	caecum	CP1802-cc475_TCTCGG	16S rDNA caecum	AMPLICON	METAGENOMIC	PCR	paired	ILLUMINA	Illumina MiSeq	PCR of V3-V4 DNA16S region	fastq	CP1802-cc475_TCTCGG-C9F6J_L001_R1.fastq.gz	CP1802-cc475_TCTCGG-C9F6J_L001_R2.fastq.gz
SRR11430026	SRP254184	PRJNA615661	SAMN14464173	476	caecum	CP1802-cc476_ATAAGA	16S rDNA caecum	AMPLICON	METAGENOMIC	PCR	paired	ILLUMINA	Illumina MiSeq	PCR of V3-V4 DNA16S region	fastq	CP1802-cc476_ATAAGA-C9F6J_L001_R1.fastq.gz	CP1802-cc476_ATAAGA-C9F6J_L001_R2.fastq.gz
SRR11430025	SRP254184	PRJNA615661	SAMN14464174	478	caecum	CP1802-cc478_ACAGTT	16S rDNA caecum	AMPLICON	METAGENOMIC	PCR	paired	ILLUMINA	Illumina MiSeq	PCR of V3-V4 DNA16S region	fastq	CP1802-cc478_ACAGTT-C9F6J_L001_R1.fastq.gz	CP1802-cc478_ACAGTT-C9F6J_L001_R2.fastq.gz
SRR11430024	SRP254184	PRJNA615661	SAMN14464175	479	caecum	CP1802-cc479_TTGCCC	16S rDNA caecum	AMPLICON	METAGENOMIC	PCR	paired	ILLUMINA	Illumina MiSeq	PCR of V3-V4 DNA16S region	fastq	CP1802-cc479_TTGCCC-C9F6J_L001_R1.fastq.gz	CP1802-cc479_TTGCCC-C9F6J_L001_R2.fastq.gz
SRR11430023	SRP254184	PRJNA615661	SAMN14464176	480	caecum	CP1802-cc480_AAATTG	16S rDNA caecum	AMPLICON	METAGENOMIC	PCR	paired	ILLUMINA	Illumina MiSeq	PCR of V3-V4 DNA16S region	fastq	CP1802-cc480_AAATTG-C9F6J_L001_R1.fastq.gz	CP1802-cc480_AAATTG-C9F6J_L001_R2.fastq.gz
SRR11430022	SRP254184	PRJNA615661	SAMN14464177	481	caecum	CP1802-cc481_TTAAAT	16S rDNA caecum	AMPLICON	METAGENOMIC	PCR	paired	ILLUMINA	Illumina MiSeq	PCR of V3-V4 DNA16S region	fastq	CP1802-cc481_TTAAAT-C9F6J_L001_R1.fastq.gz	CP1802-cc481_TTAAAT-C9F6J_L001_R2.fastq.gz
SRR11430021	SRP254184	PRJNA615661	SAMN14464178	482	caecum	CP1802-cc482_AGGTTC	16S rDNA caecum	AMPLICON	METAGENOMIC	PCR	paired	ILLUMINA	Illumina MiSeq	PCR of V3-V4 DNA16S region	fastq	CP1802-cc482_AGGTTC-C9F6J_L001_R1.fastq.gz	CP1802-cc482_AGGTTC-C9F6J_L001_R2.fastq.gz
SRR11430020	SRP254184	PRJNA615661	SAMN14464179	483	caecum	CP1802-cc483_ACTCGA	16S rDNA caecum	AMPLICON	METAGENOMIC	PCR	paired	ILLUMINA	Illumina MiSeq	PCR of V3-V4 DNA16S region	fastq	CP1802-cc483_ACTCGA-C9F6J_L001_R1.fastq.gz	CP1802-cc483_ACTCGA-C9F6J_L001_R2.fastq.gz
SRR11430019	SRP254184	PRJNA615661	SAMN14464180	484	caecum	CP1802-cc484_TTTGTA	16S rDNA caecum	AMPLICON	METAGENOMIC	PCR	paired	ILLUMINA	Illumina MiSeq	PCR of V3-V4 DNA16S region	fastq	CP1802-cc484_TTTGTA-C9F6J_L001_R1.fastq.gz	CP1802-cc484_TTTGTA-C9F6J_L001_R2.fastq.gz
SRR11430018	SRP254184	PRJNA615661	SAMN14464181	485	caecum	CP1802-cc485_CAGATG	16S rDNA caecum	AMPLICON	METAGENOMIC	PCR	paired	ILLUMINA	Illumina MiSeq	PCR of V3-V4 DNA16S region	fastq	CP1802-cc485_CAGATG-C9F6J_L001_R1.fastq.gz	CP1802-cc485_CAGATG-C9F6J_L001_R2.fastq.gz
SRR11430017	SRP254184	PRJNA615661	SAMN14464182	486	caecum	CP1802-cc486_AGCCTG	16S rDNA caecum	AMPLICON	METAGENOMIC	PCR	paired	ILLUMINA	Illumina MiSeq	PCR of V3-V4 DNA16S region	fastq	CP1802-cc486_AGCCTG-C9F6J_L001_R1.fastq.gz	CP1802-cc486_AGCCTG-C9F6J_L001_R2.fastq.gz
SRR11430015	SRP254184	PRJNA615661	SAMN14464183	488	caecum	CP1802-cc488_CATGTT	16S rDNA caecum	AMPLICON	METAGENOMIC	PCR	paired	ILLUMINA	Illumina MiSeq	PCR of V3-V4 DNA16S region	fastq	CP1802-cc488_CATGTT-C9F6J_L001_R1.fastq.gz	CP1802-cc488_CATGTT-C9F6J_L001_R2.fastq.gz
SRR11430014	SRP254184	PRJNA615661	SAMN14464184	489	caecum	CP1802-cc489_CCATTG	16S rDNA caecum	AMPLICON	METAGENOMIC	PCR	paired	ILLUMINA	Illumina MiSeq	PCR of V3-V4 DNA16S region	fastq	CP1802-cc489_CCATTG-C9F6J_L001_R1.fastq.gz	CP1802-cc489_CCATTG-C9F6J_L001_R2.fastq.gz
SRR11430013	SRP254184	PRJNA615661	SAMN14464185	491	caecum	CP1802-cc491_TATGCG	16S rDNA caecum	AMPLICON	METAGENOMIC	PCR	paired	ILLUMINA	Illumina MiSeq	PCR of V3-V4 DNA16S region	fastq	CP1802-cc491_TATGCG-C9F6J_L001_R1.fastq.gz	CP1802-cc491_TATGCG-C9F6J_L001_R2.fastq.gz
SRR11430012	SRP254184	PRJNA615661	SAMN14464186	493	caecum	CP1802-cc493_ACATAT	16S rDNA caecum	AMPLICON	METAGENOMIC	PCR	paired	ILLUMINA	Illumina MiSeq	PCR of V3-V4 DNA16S region	fastq	CP1802-cc493_ACATAT-C9F6J_L001_R1.fastq.gz	CP1802-cc493_ACATAT-C9F6J_L001_R2.fastq.gz
SRR11430011	SRP254184	PRJNA615661	SAMN14464187	494	caecum	CP1802-cc494_CAGAGC	16S rDNA caecum	AMPLICON	METAGENOMIC	PCR	paired	ILLUMINA	Illumina MiSeq	PCR of V3-V4 DNA16S region	fastq	CP1802-cc494_CAGAGC-C9F6J_L001_R1.fastq.gz	CP1802-cc494_CAGAGC-C9F6J_L001_R2.fastq.gz
SRR11430010	SRP254184	PRJNA615661	SAMN14464188	495	caecum	CP1802-cc495_AATATG	16S rDNA caecum	AMPLICON	METAGENOMIC	PCR	paired	ILLUMINA	Illumina MiSeq	PCR of V3-V4 DNA16S region	fastq	CP1802-cc495_AATATG-C9F6J_L001_R1.fastq.gz	CP1802-cc495_AATATG-C9F6J_L001_R2.fastq.gz
SRR11430009	SRP254184	PRJNA615661	SAMN14464189	497	caecum	CP1802-cc497_AATCAC	16S rDNA caecum	AMPLICON	METAGENOMIC	PCR	paired	ILLUMINA	Illumina MiSeq	PCR of V3-V4 DNA16S region	fastq	CP1802-cc497_AATCAC-C9F6J_L001_R1.fastq.gz	CP1802-cc497_AATCAC-C9F6J_L001_R2.fastq.gz
SRR11430008	SRP254184	PRJNA615661	SAMN14464190	499	caecum	CP1802-cc499_CTCTCG	16S rDNA caecum	AMPLICON	METAGENOMIC	PCR	paired	ILLUMINA	Illumina MiSeq	PCR of V3-V4 DNA16S region	fastq	CP1802-cc499_CTCTCG-C9F6J_L001_R1.fastq.gz	CP1802-cc499_CTCTCG-C9F6J_L001_R2.fastq.gz
SRR11430007	SRP254184	PRJNA615661	SAMN14464191	500	caecum	CP1802-cc500_CACACT	16S rDNA caecum	AMPLICON	METAGENOMIC	PCR	paired	ILLUMINA	Illumina MiSeq	PCR of V3-V4 DNA16S region	fastq	CP1802-cc500_CACACT-C9F6J_L001_R1.fastq.gz	CP1802-cc500_CACACT-C9F6J_L001_R2.fastq.gz
SRR11430006	SRP254184	PRJNA615661	SAMN14464192	301	appendix_vermiformis	CP1802v301_CTGAGG-C	16S rDNA caecum	AMPLICON	METAGENOMIC	PCR	paired	ILLUMINA	Illumina MiSeq	PCR of V3-V4 DNA16S region	fastq	CP1802v301_CTGAGG-CCCBN_L001_R1.fastq.gz	CP1802v301_CTGAGG-CCCBN_L001_R2.fastq.gz
SRR11430004	SRP254184	PRJNA615661	SAMN14464193	302	appendix_vermiformis	CP1802v302_TCATCA-C	16S rDNA caecum	AMPLICON	METAGENOMIC	PCR	paired	ILLUMINA	Illumina MiSeq	PCR of V3-V4 DNA16S region	fastq	CP1802v302_TCATCA-CCCBN_L001_R1.fastq.gz	CP1802v302_TCATCA-CCCBN_L001_R2.fastq.gz
SRR11430003	SRP254184	PRJNA615661	SAMN14464194	303	appendix_vermiformis	CP1802v303_CTTGCA-C	16S rDNA caecum	AMPLICON	METAGENOMIC	PCR	paired	ILLUMINA	Illumina MiSeq	PCR of V3-V4 DNA16S region	fastq	CP1802v303_CTTGCA-CCCBN_L001_R1.fastq.gz	CP1802v303_CTTGCA-CCCBN_L001_R2.fastq.gz
SRR11430002	SRP254184	PRJNA615661	SAMN14464195	304	appendix_vermiformis	CP1802v304_CATGTT-C	16S rDNA caecum	AMPLICON	METAGENOMIC	PCR	paired	ILLUMINA	Illumina MiSeq	PCR of V3-V4 DNA16S region	fastq	CP1802v304_CATGTT-CCCBN_L001_R1.fastq.gz	CP1802v304_CATGTT-CCCBN_L001_R2.fastq.gz
SRR11430001	SRP254184	PRJNA615661	SAMN14464196	309	appendix_vermiformis	CP1802v309_TTCGAG-C	16S rDNA caecum	AMPLICON	METAGENOMIC	PCR	paired	ILLUMINA	Illumina MiSeq	PCR of V3-V4 DNA16S region	fastq	CP1802v309_TTCGAG-CCCBN_L001_R1.fastq.gz	CP1802v309_TTCGAG-CCCBN_L001_R2.fastq.gz
SRR11430000	SRP254184	PRJNA615661	SAMN14464197	310	appendix_vermiformis	CP1802v310_AAGCTA-C	16S rDNA caecum	AMPLICON	METAGENOMIC	PCR	paired	ILLUMINA	Illumina MiSeq	PCR of V3-V4 DNA16S region	fastq	CP1802v310_AAGCTA-CCCBN_L001_R1.fastq.gz	CP1802v310_AAGCTA-CCCBN_L001_R2.fastq.gz
SRR11429999	SRP254184	PRJNA615661	SAMN14464198	312	appendix_vermiformis	CP1802v312_TCCCCA-C	16S rDNA caecum	AMPLICON	METAGENOMIC	PCR	paired	ILLUMINA	Illumina MiSeq	PCR of V3-V4 DNA16S region	fastq	CP1802v312_TCCCCA-CCCBN_L001_R1.fastq.gz	CP1802v312_TCCCCA-CCCBN_L001_R2.fastq.gz
SRR11429998	SRP254184	PRJNA615661	SAMN14464199	313	appendix_vermiformis	CP1802v313_AACTAG-C	16S rDNA caecum	AMPLICON	METAGENOMIC	PCR	paired	ILLUMINA	Illumina MiSeq	PCR of V3-V4 DNA16S region	fastq	CP1802v313_AACTAG-CCCBN_L001_R1.fastq.gz	CP1802v313_AACTAG-CCCBN_L001_R2.fastq.gz
SRR11429997	SRP254184	PRJNA615661	SAMN14464200	314	appendix_vermiformis	CP1802v314_GTTCGC-C	16S rDNA caecum	AMPLICON	METAGENOMIC	PCR	paired	ILLUMINA	Illumina MiSeq	PCR of V3-V4 DNA16S region	fastq	CP1802v314_GTTCGC-CCCBN_L001_R1.fastq.gz	CP1802v314_GTTCGC-CCCBN_L001_R2.fastq.gz
SRR11429996	SRP254184	PRJNA615661	SAMN14464201	316	appendix_vermiformis	CP1802v316_ATAAGA-C	16S rDNA caecum	AMPLICON	METAGENOMIC	PCR	paired	ILLUMINA	Illumina MiSeq	PCR of V3-V4 DNA16S region	fastq	CP1802v316_ATAAGA-CCCBN_L001_R1.fastq.gz	CP1802v316_ATAAGA-CCCBN_L001_R2.fastq.gz
SRR11429995	SRP254184	PRJNA615661	SAMN14464202	319	appendix_vermiformis	CP1802v319_ACAGTT-C	16S rDNA caecum	AMPLICON	METAGENOMIC	PCR	paired	ILLUMINA	Illumina MiSeq	PCR of V3-V4 DNA16S region	fastq	CP1802v319_ACAGTT-CCCBN_L001_R1.fastq.gz	CP1802v319_ACAGTT-CCCBN_L001_R2.fastq.gz
SRR11429893	SRP254184	PRJNA615661	SAMN14464203	320	appendix_vermiformis	CP1802v320_TTGCCC-C	16S rDNA caecum	AMPLICON	METAGENOMIC	PCR	paired	ILLUMINA	Illumina MiSeq	PCR of V3-V4 DNA16S region	fastq	CP1802v320_TTGCCC-CCCBN_L001_R1.fastq.gz	CP1802v320_TTGCCC-CCCBN_L001_R2.fastq.gz
SRR11429892	SRP254184	PRJNA615661	SAMN14464204	321	appendix_vermiformis	CP1802v321_CAGTCT-C	16S rDNA caecum	AMPLICON	METAGENOMIC	PCR	paired	ILLUMINA	Illumina MiSeq	PCR of V3-V4 DNA16S region	fastq	CP1802v321_CAGTCT-CCCBN_L001_R1.fastq.gz	CP1802v321_CAGTCT-CCCBN_L001_R2.fastq.gz
SRR11429891	SRP254184	PRJNA615661	SAMN14464205	322	appendix_vermiformis	CP1802v322_TTAAAT-C	16S rDNA caecum	AMPLICON	METAGENOMIC	PCR	paired	ILLUMINA	Illumina MiSeq	PCR of V3-V4 DNA16S region	fastq	CP1802v322_TTAAAT-CCCBN_L001_R1.fastq.gz	CP1802v322_TTAAAT-CCCBN_L001_R2.fastq.gz
SRR11429890	SRP254184	PRJNA615661	SAMN14464206	324	appendix_vermiformis	CP1802v324_ACTCGA-C	16S rDNA caecum	AMPLICON	METAGENOMIC	PCR	paired	ILLUMINA	Illumina MiSeq	PCR of V3-V4 DNA16S region	fastq	CP1802v324_ACTCGA-CCCBN_L001_R1.fastq.gz	CP1802v324_ACTCGA-CCCBN_L001_R2.fastq.gz
SRR11429889	SRP254184	PRJNA615661	SAMN14464207	326	appendix_vermiformis	CP1802v326_CAGATG-C	16S rDNA caecum	AMPLICON	METAGENOMIC	PCR	paired	ILLUMINA	Illumina MiSeq	PCR of V3-V4 DNA16S region	fastq	CP1802v326_CAGATG-CCCBN_L001_R1.fastq.gz	CP1802v326_CAGATG-CCCBN_L001_R2.fastq.gz
SRR11429888	SRP254184	PRJNA615661	SAMN14464208	327	appendix_vermiformis	CP1802v327_TCGCGC-C	16S rDNA caecum	AMPLICON	METAGENOMIC	PCR	paired	ILLUMINA	Illumina MiSeq	PCR of V3-V4 DNA16S region	fastq	CP1802v327_TCGCGC-CCCBN_L001_R1.fastq.gz	CP1802v327_TCGCGC-CCCBN_L001_R2.fastq.gz
SRR11429887	SRP254184	PRJNA615661	SAMN14464209	328	appendix_vermiformis	CP1802v328_TAACTT-C	16S rDNA caecum	AMPLICON	METAGENOMIC	PCR	paired	ILLUMINA	Illumina MiSeq	PCR of V3-V4 DNA16S region	fastq	CP1802v328_TAACTT-CCCBN_L001_R1.fastq.gz	CP1802v328_TAACTT-CCCBN_L001_R2.fastq.gz
SRR11429886	SRP254184	PRJNA615661	SAMN14464210	329	appendix_vermiformis	CP1802v329_CTGTAA-C	16S rDNA caecum	AMPLICON	METAGENOMIC	PCR	paired	ILLUMINA	Illumina MiSeq	PCR of V3-V4 DNA16S region	fastq	CP1802v329_CTGTAA-CCCBN_L001_R1.fastq.gz	CP1802v329_CTGTAA-CCCBN_L001_R2.fastq.gz
SRR11429885	SRP254184	PRJNA615661	SAMN14464211	330	appendix_vermiformis	CP1802v330_CCATTG-C	16S rDNA caecum	AMPLICON	METAGENOMIC	PCR	paired	ILLUMINA	Illumina MiSeq	PCR of V3-V4 DNA16S region	fastq	CP1802v330_CCATTG-CCCBN_L001_R1.fastq.gz	CP1802v330_CCATTG-CCCBN_L001_R2.fastq.gz
SRR11429884	SRP254184	PRJNA615661	SAMN14464212	331	appendix_vermiformis	CP1802v331_TAGGCT-C	16S rDNA caecum	AMPLICON	METAGENOMIC	PCR	paired	ILLUMINA	Illumina MiSeq	PCR of V3-V4 DNA16S region	fastq	CP1802v331_TAGGCT-CCCBN_L001_R1.fastq.gz	CP1802v331_TAGGCT-CCCBN_L001_R2.fastq.gz
SRR11429882	SRP254184	PRJNA615661	SAMN14464213	332	appendix_vermiformis	CP1802v332_TTCTTG-C	16S rDNA caecum	AMPLICON	METAGENOMIC	PCR	paired	ILLUMINA	Illumina MiSeq	PCR of V3-V4 DNA16S region	fastq	CP1802v332_TTCTTG-CCCBN_L001_R1.fastq.gz	CP1802v332_TTCTTG-CCCBN_L001_R2.fastq.gz
SRR11429881	SRP254184	PRJNA615661	SAMN14464214	333	appendix_vermiformis	CP1802v333_CCGACC-C	16S rDNA caecum	AMPLICON	METAGENOMIC	PCR	paired	ILLUMINA	Illumina MiSeq	PCR of V3-V4 DNA16S region	fastq	CP1802v333_CCGACC-CCCBN_L001_R1.fastq.gz	CP1802v333_CCGACC-CCCBN_L001_R2.fastq.gz
SRR11429880	SRP254184	PRJNA615661	SAMN14464215	334	appendix_vermiformis	CP1802v334_TTAGCT-C	16S rDNA caecum	AMPLICON	METAGENOMIC	PCR	paired	ILLUMINA	Illumina MiSeq	PCR of V3-V4 DNA16S region	fastq	CP1802v334_TTAGCT-CCCBN_L001_R1.fastq.gz	CP1802v334_TTAGCT-CCCBN_L001_R2.fastq.gz
SRR11429879	SRP254184	PRJNA615661	SAMN14464216	335	appendix_vermiformis	CP1802v335_CAGAGC-C	16S rDNA caecum	AMPLICON	METAGENOMIC	PCR	paired	ILLUMINA	Illumina MiSeq	PCR of V3-V4 DNA16S region	fastq	CP1802v335_CAGAGC-CCCBN_L001_R1.fastq.gz	CP1802v335_CAGAGC-CCCBN_L001_R2.fastq.gz
SRR11429878	SRP254184	PRJNA615661	SAMN14464217	337	appendix_vermiformis	CP1802v337_ATTCTC-C	16S rDNA caecum	AMPLICON	METAGENOMIC	PCR	paired	ILLUMINA	Illumina MiSeq	PCR of V3-V4 DNA16S region	fastq	CP1802v337_ATTCTC-CCCBN_L001_R1.fastq.gz	CP1802v337_ATTCTC-CCCBN_L001_R2.fastq.gz
SRR11429877	SRP254184	PRJNA615661	SAMN14464218	338	appendix_vermiformis	CP1802v338_CGAAGG-C	16S rDNA caecum	AMPLICON	METAGENOMIC	PCR	paired	ILLUMINA	Illumina MiSeq	PCR of V3-V4 DNA16S region	fastq	CP1802v338_CGAAGG-CCCBN_L001_R1.fastq.gz	CP1802v338_CGAAGG-CCCBN_L001_R2.fastq.gz
SRR11429876	SRP254184	PRJNA615661	SAMN14464219	340	appendix_vermiformis	CP1802v340_AAAGTA-C	16S rDNA caecum	AMPLICON	METAGENOMIC	PCR	paired	ILLUMINA	Illumina MiSeq	PCR of V3-V4 DNA16S region	fastq	CP1802v340_AAAGTA-CCCBN_L001_R1.fastq.gz	CP1802v340_AAAGTA-CCCBN_L001_R2.fastq.gz
SRR11429875	SRP254184	PRJNA615661	SAMN14464220	341	appendix_vermiformis	CP1802v341_TCAGCG-C	16S rDNA caecum	AMPLICON	METAGENOMIC	PCR	paired	ILLUMINA	Illumina MiSeq	PCR of V3-V4 DNA16S region	fastq	CP1802v341_TCAGCG-CCCBN_L001_R1.fastq.gz	CP1802v341_TCAGCG-CCCBN_L001_R2.fastq.gz
SRR11429874	SRP254184	PRJNA615661	SAMN14464221	342	appendix_vermiformis	CP1802v342_AGGCGC-C	16S rDNA caecum	AMPLICON	METAGENOMIC	PCR	paired	ILLUMINA	Illumina MiSeq	PCR of V3-V4 DNA16S region	fastq	CP1802v342_AGGCGC-CCCBN_L001_R1.fastq.gz	CP1802v342_AGGCGC-CCCBN_L001_R2.fastq.gz
SRR11429873	SRP254184	PRJNA615661	SAMN14464222	343	appendix_vermiformis	CP1802v343_AATCCG-C	16S rDNA caecum	AMPLICON	METAGENOMIC	PCR	paired	ILLUMINA	Illumina MiSeq	PCR of V3-V4 DNA16S region	fastq	CP1802v343_AATCCG-CCCBN_L001_R1.fastq.gz	CP1802v343_AATCCG-CCCBN_L001_R2.fastq.gz
SRR11429871	SRP254184	PRJNA615661	SAMN14464223	344	appendix_vermiformis	CP1802v344_TGATGC-C	16S rDNA caecum	AMPLICON	METAGENOMIC	PCR	paired	ILLUMINA	Illumina MiSeq	PCR of V3-V4 DNA16S region	fastq	CP1802v344_TGATGC-CCCBN_L001_R1.fastq.gz	CP1802v344_TGATGC-CCCBN_L001_R2.fastq.gz
SRR11429870	SRP254184	PRJNA615661	SAMN14464224	347	appendix_vermiformis	CP1802v347_GTGAAT-C	16S rDNA caecum	AMPLICON	METAGENOMIC	PCR	paired	ILLUMINA	Illumina MiSeq	PCR of V3-V4 DNA16S region	fastq	CP1802v347_GTGAAT-CCCBN_L001_R1.fastq.gz	CP1802v347_GTGAAT-CCCBN_L001_R2.fastq.gz
SRR11429869	SRP254184	PRJNA615661	SAMN14464225	349	appendix_vermiformis	CP1802v349_TGTCGT-C	16S rDNA caecum	AMPLICON	METAGENOMIC	PCR	paired	ILLUMINA	Illumina MiSeq	PCR of V3-V4 DNA16S region	fastq	CP1802v349_TGTCGT-CCCBN_L001_R1.fastq.gz	CP1802v349_TGTCGT-CCCBN_L001_R2.fastq.gz
SRR11429868	SRP254184	PRJNA615661	SAMN14464226	350	appendix_vermiformis	CP1802v350_CACTAA-C	16S rDNA caecum	AMPLICON	METAGENOMIC	PCR	paired	ILLUMINA	Illumina MiSeq	PCR of V3-V4 DNA16S region	fastq	CP1802v350_CACTAA-CCCBN_L001_R1.fastq.gz	CP1802v350_CACTAA-CCCBN_L001_R2.fastq.gz
SRR11429867	SRP254184	PRJNA615661	SAMN14464227	352	appendix_vermiformis	CP1802v352_ACATTA-C	16S rDNA caecum	AMPLICON	METAGENOMIC	PCR	paired	ILLUMINA	Illumina MiSeq	PCR of V3-V4 DNA16S region	fastq	CP1802v352_ACATTA-CCCBN_L001_R1.fastq.gz	CP1802v352_ACATTA-CCCBN_L001_R2.fastq.gz
SRR11429866	SRP254184	PRJNA615661	SAMN14464228	353	appendix_vermiformis	CP1802v353_GGGTCT-C	16S rDNA caecum	AMPLICON	METAGENOMIC	PCR	paired	ILLUMINA	Illumina MiSeq	PCR of V3-V4 DNA16S region	fastq	CP1802v353_GGGTCT-CCCBN_L001_R1.fastq.gz	CP1802v353_GGGTCT-CCCBN_L001_R2.fastq.gz
SRR11429865	SRP254184	PRJNA615661	SAMN14464229	354	appendix_vermiformis	CP1802v354_CCAAGC-C	16S rDNA caecum	AMPLICON	METAGENOMIC	PCR	paired	ILLUMINA	Illumina MiSeq	PCR of V3-V4 DNA16S region	fastq	CP1802v354_CCAAGC-CCCBN_L001_R1.fastq.gz	CP1802v354_CCAAGC-CCCBN_L001_R2.fastq.gz
SRR11429864	SRP254184	PRJNA615661	SAMN14464230	356	appendix_vermiformis	CP1802v356_CGTTAA-C	16S rDNA caecum	AMPLICON	METAGENOMIC	PCR	paired	ILLUMINA	Illumina MiSeq	PCR of V3-V4 DNA16S region	fastq	CP1802v356_CGTTAA-CCCBN_L001_R1.fastq.gz	CP1802v356_CGTTAA-CCCBN_L001_R2.fastq.gz
SRR11429863	SRP254184	PRJNA615661	SAMN14464231	357	appendix_vermiformis	CP1802v357_GTGTAG-C	16S rDNA caecum	AMPLICON	METAGENOMIC	PCR	paired	ILLUMINA	Illumina MiSeq	PCR of V3-V4 DNA16S region	fastq	CP1802v357_GTGTAG-CCCBN_L001_R1.fastq.gz	CP1802v357_GTGTAG-CCCBN_L001_R2.fastq.gz
SRR11429862	SRP254184	PRJNA615661	SAMN14464232	358	appendix_vermiformis	CP1802v358_CACCTC-C	16S rDNA caecum	AMPLICON	METAGENOMIC	PCR	paired	ILLUMINA	Illumina MiSeq	PCR of V3-V4 DNA16S region	fastq	CP1802v358_CACCTC-CCCBN_L001_R1.fastq.gz	CP1802v358_CACCTC-CCCBN_L001_R2.fastq.gz
SRR11429860	SRP254184	PRJNA615661	SAMN14464233	359	appendix_vermiformis	CP1802v359_TAAATG-C	16S rDNA caecum	AMPLICON	METAGENOMIC	PCR	paired	ILLUMINA	Illumina MiSeq	PCR of V3-V4 DNA16S region	fastq	CP1802v359_TAAATG-CCCBN_L001_R1.fastq.gz	CP1802v359_TAAATG-CCCBN_L001_R2.fastq.gz
SRR11429859	SRP254184	PRJNA615661	SAMN14464234	360	appendix_vermiformis	CP1802v360_CTTGAC-C	16S rDNA caecum	AMPLICON	METAGENOMIC	PCR	paired	ILLUMINA	Illumina MiSeq	PCR of V3-V4 DNA16S region	fastq	CP1802v360_CTTGAC-CCCBN_L001_R1.fastq.gz	CP1802v360_CTTGAC-CCCBN_L001_R2.fastq.gz
SRR11429858	SRP254184	PRJNA615661	SAMN14464235	362	appendix_vermiformis	CP1802v362_AAGTAA-C	16S rDNA caecum	AMPLICON	METAGENOMIC	PCR	paired	ILLUMINA	Illumina MiSeq	PCR of V3-V4 DNA16S region	fastq	CP1802v362_AAGTAA-CCCBN_L001_R1.fastq.gz	CP1802v362_AAGTAA-CCCBN_L001_R2.fastq.gz
SRR11429857	SRP254184	PRJNA615661	SAMN14464236	364	appendix_vermiformis	CP1802v364_AGAATC-C	16S rDNA caecum	AMPLICON	METAGENOMIC	PCR	paired	ILLUMINA	Illumina MiSeq	PCR of V3-V4 DNA16S region	fastq	CP1802v364_AGAATC-CCCBN_L001_R1.fastq.gz	CP1802v364_AGAATC-CCCBN_L001_R2.fastq.gz
SRR11429856	SRP254184	PRJNA615661	SAMN14464237	366	appendix_vermiformis	CP1802v366_CGGAAC-C	16S rDNA caecum	AMPLICON	METAGENOMIC	PCR	paired	ILLUMINA	Illumina MiSeq	PCR of V3-V4 DNA16S region	fastq	CP1802v366_CGGAAC-CCCBN_L001_R1.fastq.gz	CP1802v366_CGGAAC-CCCBN_L001_R2.fastq.gz
SRR11429855	SRP254184	PRJNA615661	SAMN14464238	367	appendix_vermiformis	CP1802v367_ATTTGT-C	16S rDNA caecum	AMPLICON	METAGENOMIC	PCR	paired	ILLUMINA	Illumina MiSeq	PCR of V3-V4 DNA16S region	fastq	CP1802v367_ATTTGT-CCCBN_L001_R1.fastq.gz	CP1802v367_ATTTGT-CCCBN_L001_R2.fastq.gz
SRR11429854	SRP254184	PRJNA615661	SAMN14464239	368	appendix_vermiformis	CP1802v368_GCCCAC-C	16S rDNA caecum	AMPLICON	METAGENOMIC	PCR	paired	ILLUMINA	Illumina MiSeq	PCR of V3-V4 DNA16S region	fastq	CP1802v368_GCCCAC-CCCBN_L001_R1.fastq.gz	CP1802v368_GCCCAC-CCCBN_L001_R2.fastq.gz
SRR11429853	SRP254184	PRJNA615661	SAMN14464240	369	appendix_vermiformis	CP1802v369_AACCAT-C	16S rDNA caecum	AMPLICON	METAGENOMIC	PCR	paired	ILLUMINA	Illumina MiSeq	PCR of V3-V4 DNA16S region	fastq	CP1802v369_AACCAT-CCCBN_L001_R1.fastq.gz	CP1802v369_AACCAT-CCCBN_L001_R2.fastq.gz
SRR11429852	SRP254184	PRJNA615661	SAMN14464241	370	appendix_vermiformis	CP1802v370_GGGGGA-C	16S rDNA caecum	AMPLICON	METAGENOMIC	PCR	paired	ILLUMINA	Illumina MiSeq	PCR of V3-V4 DNA16S region	fastq	CP1802v370_GGGGGA-CCCBN_L001_R1.fastq.gz	CP1802v370_GGGGGA-CCCBN_L001_R2.fastq.gz
SRR11429851	SRP254184	PRJNA615661	SAMN14464242	371	appendix_vermiformis	CP1802v371_TAGTAC-C	16S rDNA caecum	AMPLICON	METAGENOMIC	PCR	paired	ILLUMINA	Illumina MiSeq	PCR of V3-V4 DNA16S region	fastq	CP1802v371_TAGTAC-CCCBN_L001_R1.fastq.gz	CP1802v371_TAGTAC-CCCBN_L001_R2.fastq.gz
SRR11429848	SRP254184	PRJNA615661	SAMN14464243	372	appendix_vermiformis	CP1802v372_GTTCTT-C	16S rDNA caecum	AMPLICON	METAGENOMIC	PCR	paired	ILLUMINA	Illumina MiSeq	PCR of V3-V4 DNA16S region	fastq	CP1802v372_GTTCTT-CCCBN_L001_R1.fastq.gz	CP1802v372_GTTCTT-CCCBN_L001_R2.fastq.gz
SRR11429847	SRP254184	PRJNA615661	SAMN14464244	373	appendix_vermiformis	CP1802v373_ACAGAC-C	16S rDNA caecum	AMPLICON	METAGENOMIC	PCR	paired	ILLUMINA	Illumina MiSeq	PCR of V3-V4 DNA16S region	fastq	CP1802v373_ACAGAC-CCCBN_L001_R1.fastq.gz	CP1802v373_ACAGAC-CCCBN_L001_R2.fastq.gz
SRR11429846	SRP254184	PRJNA615661	SAMN14464245	374	appendix_vermiformis	CP1802v374_GTTGCG-C	16S rDNA caecum	AMPLICON	METAGENOMIC	PCR	paired	ILLUMINA	Illumina MiSeq	PCR of V3-V4 DNA16S region	fastq	CP1802v374_GTTGCG-CCCBN_L001_R1.fastq.gz	CP1802v374_GTTGCG-CCCBN_L001_R2.fastq.gz
SRR11429845	SRP254184	PRJNA615661	SAMN14464246	375	appendix_vermiformis	CP1802v375_AAACTC-C	16S rDNA caecum	AMPLICON	METAGENOMIC	PCR	paired	ILLUMINA	Illumina MiSeq	PCR of V3-V4 DNA16S region	fastq	CP1802v375_AAACTC-CCCBN_L001_R1.fastq.gz	CP1802v375_AAACTC-CCCBN_L001_R2.fastq.gz
SRR11429844	SRP254184	PRJNA615661	SAMN14464247	377	appendix_vermiformis	CP1802v377_TCTTAA-C	16S rDNA caecum	AMPLICON	METAGENOMIC	PCR	paired	ILLUMINA	Illumina MiSeq	PCR of V3-V4 DNA16S region	fastq	CP1802v377_TCTTAA-CCCBN_L001_R1.fastq.gz	CP1802v377_TCTTAA-CCCBN_L001_R2.fastq.gz
SRR11429843	SRP254184	PRJNA615661	SAMN14464248	378	appendix_vermiformis	CP1802v378_CGCGTG-C	16S rDNA caecum	AMPLICON	METAGENOMIC	PCR	paired	ILLUMINA	Illumina MiSeq	PCR of V3-V4 DNA16S region	fastq	CP1802v378_CGCGTG-CCCBN_L001_R1.fastq.gz	CP1802v378_CGCGTG-CCCBN_L001_R2.fastq.gz
SRR11429842	SRP254184	PRJNA615661	SAMN14464249	380	appendix_vermiformis	CP1802v380_AAGGCC-C	16S rDNA caecum	AMPLICON	METAGENOMIC	PCR	paired	ILLUMINA	Illumina MiSeq	PCR of V3-V4 DNA16S region	fastq	CP1802v380_AAGGCC-CCCBN_L001_R1.fastq.gz	CP1802v380_AAGGCC-CCCBN_L001_R2.fastq.gz
SRR11429841	SRP254184	PRJNA615661	SAMN14464250	381	appendix_vermiformis	CP1802v381_TGCACG-C	16S rDNA caecum	AMPLICON	METAGENOMIC	PCR	paired	ILLUMINA	Illumina MiSeq	PCR of V3-V4 DNA16S region	fastq	CP1802v381_TGCACG-CCCBN_L001_R1.fastq.gz	CP1802v381_TGCACG-CCCBN_L001_R2.fastq.gz
SRR11429840	SRP254184	PRJNA615661	SAMN14464251	382	appendix_vermiformis	CP1802v382_CAGTGA-C	16S rDNA caecum	AMPLICON	METAGENOMIC	PCR	paired	ILLUMINA	Illumina MiSeq	PCR of V3-V4 DNA16S region	fastq	CP1802v382_CAGTGA-CCCBN_L001_R1.fastq.gz	CP1802v382_CAGTGA-CCCBN_L001_R2.fastq.gz
SRR11429839	SRP254184	PRJNA615661	SAMN14464252	383	appendix_vermiformis	CP1802v383_GCCGGT-C	16S rDNA caecum	AMPLICON	METAGENOMIC	PCR	paired	ILLUMINA	Illumina MiSeq	PCR of V3-V4 DNA16S region	fastq	CP1802v383_GCCGGT-CCCBN_L001_R1.fastq.gz	CP1802v383_GCCGGT-CCCBN_L001_R2.fastq.gz
SRR11429837	SRP254184	PRJNA615661	SAMN14464253	384	appendix_vermiformis	CP1802v384_CTTAAA-C	16S rDNA caecum	AMPLICON	METAGENOMIC	PCR	paired	ILLUMINA	Illumina MiSeq	PCR of V3-V4 DNA16S region	fastq	CP1802v384_CTTAAA-CCCBN_L001_R1.fastq.gz	CP1802v384_CTTAAA-CCCBN_L001_R2.fastq.gz
SRR11429836	SRP254184	PRJNA615661	SAMN14464254	387	appendix_vermiformis	CP1802v387_GAGCAA-C	16S rDNA caecum	AMPLICON	METAGENOMIC	PCR	paired	ILLUMINA	Illumina MiSeq	PCR of V3-V4 DNA16S region	fastq	CP1802v387_GAGCAA-CCCBN_L001_R1.fastq.gz	CP1802v387_GAGCAA-CCCBN_L001_R2.fastq.gz
SRR11429835	SRP254184	PRJNA615661	SAMN14464255	389	appendix_vermiformis	CP1802v389_GGCCTC-C	16S rDNA caecum	AMPLICON	METAGENOMIC	PCR	paired	ILLUMINA	Illumina MiSeq	PCR of V3-V4 DNA16S region	fastq	CP1802v389_GGCCTC-CCCBN_L001_R1.fastq.gz	CP1802v389_GGCCTC-CCCBN_L001_R2.fastq.gz
SRR11429834	SRP254184	PRJNA615661	SAMN14464256	390	appendix_vermiformis	CP1802v390_ATCGGG-C	16S rDNA caecum	AMPLICON	METAGENOMIC	PCR	paired	ILLUMINA	Illumina MiSeq	PCR of V3-V4 DNA16S region	fastq	CP1802v390_ATCGGG-CCCBN_L001_R1.fastq.gz	CP1802v390_ATCGGG-CCCBN_L001_R2.fastq.gz
SRR11429833	SRP254184	PRJNA615661	SAMN14464257	392	appendix_vermiformis	CP1802v392_TACTCA-C	16S rDNA caecum	AMPLICON	METAGENOMIC	PCR	paired	ILLUMINA	Illumina MiSeq	PCR of V3-V4 DNA16S region	fastq	CP1802v392_TACTCA-CCCBN_L001_R1.fastq.gz	CP1802v392_TACTCA-CCCBN_L001_R2.fastq.gz
SRR11429832	SRP254184	PRJNA615661	SAMN14464258	393	appendix_vermiformis	CP1802v393_ATAACG-C	16S rDNA caecum	AMPLICON	METAGENOMIC	PCR	paired	ILLUMINA	Illumina MiSeq	PCR of V3-V4 DNA16S region	fastq	CP1802v393_ATAACG-CCCBN_L001_R1.fastq.gz	CP1802v393_ATAACG-CCCBN_L001_R2.fastq.gz
SRR11429831	SRP254184	PRJNA615661	SAMN14464259	394	appendix_vermiformis	CP1802v394_GAGGGC-C	16S rDNA caecum	AMPLICON	METAGENOMIC	PCR	paired	ILLUMINA	Illumina MiSeq	PCR of V3-V4 DNA16S region	fastq	CP1802v394_GAGGGC-CCCBN_L001_R1.fastq.gz	CP1802v394_GAGGGC-CCCBN_L001_R2.fastq.gz
SRR11429994	SRP254184	PRJNA615661	SAMN14464260	396	appendix_vermiformis	CP1802v396_CGTTTC-C	16S rDNA caecum	AMPLICON	METAGENOMIC	PCR	paired	ILLUMINA	Illumina MiSeq	PCR of V3-V4 DNA16S region	fastq	CP1802v396_CGTTTC-CCCBN_L001_R1.fastq.gz	CP1802v396_CGTTTC-CCCBN_L001_R2.fastq.gz
SRR11429993	SRP254184	PRJNA615661	SAMN14464261	397	appendix_vermiformis	CP1802v397_CCCGTT-C	16S rDNA caecum	AMPLICON	METAGENOMIC	PCR	paired	ILLUMINA	Illumina MiSeq	PCR of V3-V4 DNA16S region	fastq	CP1802v397_CCCGTT-CCCBN_L001_R1.fastq.gz	CP1802v397_CCCGTT-CCCBN_L001_R2.fastq.gz
SRR11429992	SRP254184	PRJNA615661	SAMN14464262	398	appendix_vermiformis	CP1802v398_GGTCAC-C	16S rDNA caecum	AMPLICON	METAGENOMIC	PCR	paired	ILLUMINA	Illumina MiSeq	PCR of V3-V4 DNA16S region	fastq	CP1802v398_GGTCAC-CCCBN_L001_R1.fastq.gz	CP1802v398_GGTCAC-CCCBN_L001_R2.fastq.gz
SRR11429990	SRP254184	PRJNA615661	SAMN14464263	399	appendix_vermiformis	CP1802v399_AGTGCT-C	16S rDNA caecum	AMPLICON	METAGENOMIC	PCR	paired	ILLUMINA	Illumina MiSeq	PCR of V3-V4 DNA16S region	fastq	CP1802v399_AGTGCT-CCCBN_L001_R1.fastq.gz	CP1802v399_AGTGCT-CCCBN_L001_R2.fastq.gz
SRR11429989	SRP254184	PRJNA615661	SAMN14464264	400	appendix_vermiformis	CP1802v400_GACATC-C	16S rDNA caecum	AMPLICON	METAGENOMIC	PCR	paired	ILLUMINA	Illumina MiSeq	PCR of V3-V4 DNA16S region	fastq	CP1802v400_GACATC-CCCBN_L001_R1.fastq.gz	CP1802v400_GACATC-CCCBN_L001_R2.fastq.gz
SRR11429988	SRP254184	PRJNA615661	SAMN14464265	402	appendix_vermiformis	CP1802v402_CTCGGT-C	16S rDNA caecum	AMPLICON	METAGENOMIC	PCR	paired	ILLUMINA	Illumina MiSeq	PCR of V3-V4 DNA16S region	fastq	CP1802v402_CTCGGT-CCCBN_L001_R1.fastq.gz	CP1802v402_CTCGGT-CCCBN_L001_R2.fastq.gz
SRR11429987	SRP254184	PRJNA615661	SAMN14464266	404	appendix_vermiformis	CP1802v404_GTGTTT-C	16S rDNA caecum	AMPLICON	METAGENOMIC	PCR	paired	ILLUMINA	Illumina MiSeq	PCR of V3-V4 DNA16S region	fastq	CP1802v404_GTGTTT-CCCBN_L001_R1.fastq.gz	CP1802v404_GTGTTT-CCCBN_L001_R2.fastq.gz
SRR11429986	SRP254184	PRJNA615661	SAMN14464267	406	appendix_vermiformis	CP1802v406_ACTTTT-C	16S rDNA caecum	AMPLICON	METAGENOMIC	PCR	paired	ILLUMINA	Illumina MiSeq	PCR of V3-V4 DNA16S region	fastq	CP1802v406_ACTTTT-CCCBN_L001_R1.fastq.gz	CP1802v406_ACTTTT-CCCBN_L001_R2.fastq.gz
SRR11429985	SRP254184	PRJNA615661	SAMN14464268	407	appendix_vermiformis	CP1802v407_GGCCAA-C	16S rDNA caecum	AMPLICON	METAGENOMIC	PCR	paired	ILLUMINA	Illumina MiSeq	PCR of V3-V4 DNA16S region	fastq	CP1802v407_GGCCAA-CCCBN_L001_R1.fastq.gz	CP1802v407_GGCCAA-CCCBN_L001_R2.fastq.gz
SRR11429984	SRP254184	PRJNA615661	SAMN14464269	408	appendix_vermiformis	CP1802v408_GACAGT-C	16S rDNA caecum	AMPLICON	METAGENOMIC	PCR	paired	ILLUMINA	Illumina MiSeq	PCR of V3-V4 DNA16S region	fastq	CP1802v408_GACAGT-CCCBN_L001_R1.fastq.gz	CP1802v408_GACAGT-CCCBN_L001_R2.fastq.gz
SRR11429983	SRP254184	PRJNA615661	SAMN14464270	409	appendix_vermiformis	CP1802v409_ATGTCA-C	16S rDNA caecum	AMPLICON	METAGENOMIC	PCR	paired	ILLUMINA	Illumina MiSeq	PCR of V3-V4 DNA16S region	fastq	CP1802v409_ATGTCA-CCCBN_L001_R1.fastq.gz	CP1802v409_ATGTCA-CCCBN_L001_R2.fastq.gz
SRR11429982	SRP254184	PRJNA615661	SAMN14464271	410	appendix_vermiformis	CP1802v410_GCAGCT-C	16S rDNA caecum	AMPLICON	METAGENOMIC	PCR	paired	ILLUMINA	Illumina MiSeq	PCR of V3-V4 DNA16S region	fastq	CP1802v410_GCAGCT-CCCBN_L001_R1.fastq.gz	CP1802v410_GCAGCT-CCCBN_L001_R2.fastq.gz
SRR11429981	SRP254184	PRJNA615661	SAMN14464272	411	appendix_vermiformis	CP1802v411_CGTCGC-C	16S rDNA caecum	AMPLICON	METAGENOMIC	PCR	paired	ILLUMINA	Illumina MiSeq	PCR of V3-V4 DNA16S region	fastq	CP1802v411_CGTCGC-CCCBN_L001_R1.fastq.gz	CP1802v411_CGTCGC-CCCBN_L001_R2.fastq.gz
SRR11429979	SRP254184	PRJNA615661	SAMN14464273	412	appendix_vermiformis	CP1802v412_CGTTGG-C	16S rDNA caecum	AMPLICON	METAGENOMIC	PCR	paired	ILLUMINA	Illumina MiSeq	PCR of V3-V4 DNA16S region	fastq	CP1802v412_CGTTGG-CCCBN_L001_R1.fastq.gz	CP1802v412_CGTTGG-CCCBN_L001_R2.fastq.gz
SRR11429978	SRP254184	PRJNA615661	SAMN14464274	413	appendix_vermiformis	CP1802v413_ACCAGG-C	16S rDNA caecum	AMPLICON	METAGENOMIC	PCR	paired	ILLUMINA	Illumina MiSeq	PCR of V3-V4 DNA16S region	fastq	CP1802v413_ACCAGG-CCCBN_L001_R1.fastq.gz	CP1802v413_ACCAGG-CCCBN_L001_R2.fastq.gz
SRR11429977	SRP254184	PRJNA615661	SAMN14464275	414	appendix_vermiformis	CP1802v414_TGTGCC-C	16S rDNA caecum	AMPLICON	METAGENOMIC	PCR	paired	ILLUMINA	Illumina MiSeq	PCR of V3-V4 DNA16S region	fastq	CP1802v414_TGTGCC-CCCBN_L001_R1.fastq.gz	CP1802v414_TGTGCC-CCCBN_L001_R2.fastq.gz
SRR11429976	SRP254184	PRJNA615661	SAMN14464276	415	appendix_vermiformis	CP1802v415_GGACTT-C	16S rDNA caecum	AMPLICON	METAGENOMIC	PCR	paired	ILLUMINA	Illumina MiSeq	PCR of V3-V4 DNA16S region	fastq	CP1802v415_GGACTT-CCCBN_L001_R1.fastq.gz	CP1802v415_GGACTT-CCCBN_L001_R2.fastq.gz
SRR11429975	SRP254184	PRJNA615661	SAMN14464277	417	appendix_vermiformis	CP1802v417_CATCCT-C	16S rDNA caecum	AMPLICON	METAGENOMIC	PCR	paired	ILLUMINA	Illumina MiSeq	PCR of V3-V4 DNA16S region	fastq	CP1802v417_CATCCT-CCCBN_L001_R1.fastq.gz	CP1802v417_CATCCT-CCCBN_L001_R2.fastq.gz
SRR11429974	SRP254184	PRJNA615661	SAMN14464278	418	appendix_vermiformis	CP1802v418_GTCGGC-C	16S rDNA caecum	AMPLICON	METAGENOMIC	PCR	paired	ILLUMINA	Illumina MiSeq	PCR of V3-V4 DNA16S region	fastq	CP1802v418_GTCGGC-CCCBN_L001_R1.fastq.gz	CP1802v418_GTCGGC-CCCBN_L001_R2.fastq.gz
SRR11429973	SRP254184	PRJNA615661	SAMN14464279	420	appendix_vermiformis	CP1802v420_CAGCGT-C	16S rDNA caecum	AMPLICON	METAGENOMIC	PCR	paired	ILLUMINA	Illumina MiSeq	PCR of V3-V4 DNA16S region	fastq	CP1802v420_CAGCGT-CCCBN_L001_R1.fastq.gz	CP1802v420_CAGCGT-CCCBN_L001_R2.fastq.gz
SRR11429972	SRP254184	PRJNA615661	SAMN14464280	421	appendix_vermiformis	CP1802v421_GGATCA-C	16S rDNA caecum	AMPLICON	METAGENOMIC	PCR	paired	ILLUMINA	Illumina MiSeq	PCR of V3-V4 DNA16S region	fastq	CP1802v421_GGATCA-CCCBN_L001_R1.fastq.gz	CP1802v421_GGATCA-CCCBN_L001_R2.fastq.gz
SRR11429971	SRP254184	PRJNA615661	SAMN14464281	422	appendix_vermiformis	CP1802v422_CCCCAT-C	16S rDNA caecum	AMPLICON	METAGENOMIC	PCR	paired	ILLUMINA	Illumina MiSeq	PCR of V3-V4 DNA16S region	fastq	CP1802v422_CCCCAT-CCCBN_L001_R1.fastq.gz	CP1802v422_CCCCAT-CCCBN_L001_R2.fastq.gz
SRR11429970	SRP254184	PRJNA615661	SAMN14464282	423	appendix_vermiformis	CP1802v423_TTGTGA-C	16S rDNA caecum	AMPLICON	METAGENOMIC	PCR	paired	ILLUMINA	Illumina MiSeq	PCR of V3-V4 DNA16S region	fastq	CP1802v423_TTGTGA-CCCBN_L001_R1.fastq.gz	CP1802v423_TTGTGA-CCCBN_L001_R2.fastq.gz
SRR11429968	SRP254184	PRJNA615661	SAMN14464283	424	appendix_vermiformis	CP1802v424_AGATAG-C	16S rDNA caecum	AMPLICON	METAGENOMIC	PCR	paired	ILLUMINA	Illumina MiSeq	PCR of V3-V4 DNA16S region	fastq	CP1802v424_AGATAG-CCCBN_L001_R1.fastq.gz	CP1802v424_AGATAG-CCCBN_L001_R2.fastq.gz
SRR11429967	SRP254184	PRJNA615661	SAMN14464284	427	appendix_vermiformis	CP1802v427_ATTAGG-C	16S rDNA caecum	AMPLICON	METAGENOMIC	PCR	paired	ILLUMINA	Illumina MiSeq	PCR of V3-V4 DNA16S region	fastq	CP1802v427_ATTAGG-CCCBN_L001_R1.fastq.gz	CP1802v427_ATTAGG-CCCBN_L001_R2.fastq.gz
SRR11429966	SRP254184	PRJNA615661	SAMN14464285	429	appendix_vermiformis	CP1802v429_AAAGCG-C	16S rDNA caecum	AMPLICON	METAGENOMIC	PCR	paired	ILLUMINA	Illumina MiSeq	PCR of V3-V4 DNA16S region	fastq	CP1802v429_AAAGCG-CCCBN_L001_R1.fastq.gz	CP1802v429_AAAGCG-CCCBN_L001_R2.fastq.gz
SRR11429965	SRP254184	PRJNA615661	SAMN14464286	430	appendix_vermiformis	CP1802v430_TTGCTA-C	16S rDNA caecum	AMPLICON	METAGENOMIC	PCR	paired	ILLUMINA	Illumina MiSeq	PCR of V3-V4 DNA16S region	fastq	CP1802v430_TTGCTA-CCCBN_L001_R1.fastq.gz	CP1802v430_TTGCTA-CCCBN_L001_R2.fastq.gz
SRR11429964	SRP254184	PRJNA615661	SAMN14464287	432	appendix_vermiformis	CP1802v432_CTACTA-C	16S rDNA caecum	AMPLICON	METAGENOMIC	PCR	paired	ILLUMINA	Illumina MiSeq	PCR of V3-V4 DNA16S region	fastq	CP1802v432_CTACTA-CCCBN_L001_R1.fastq.gz	CP1802v432_CTACTA-CCCBN_L001_R2.fastq.gz
SRR11429963	SRP254184	PRJNA615661	SAMN14464288	433	appendix_vermiformis	CP1802v433_TGCGCT-C	16S rDNA caecum	AMPLICON	METAGENOMIC	PCR	paired	ILLUMINA	Illumina MiSeq	PCR of V3-V4 DNA16S region	fastq	CP1802v433_TGCGCT-CCCBN_L001_R1.fastq.gz	CP1802v433_TGCGCT-CCCBN_L001_R2.fastq.gz
SRR11429962	SRP254184	PRJNA615661	SAMN14464289	434	appendix_vermiformis	CP1802v434_ACGATC-C	16S rDNA caecum	AMPLICON	METAGENOMIC	PCR	paired	ILLUMINA	Illumina MiSeq	PCR of V3-V4 DNA16S region	fastq	CP1802v434_ACGATC-CCCBN_L001_R1.fastq.gz	CP1802v434_ACGATC-CCCBN_L001_R2.fastq.gz
SRR11429961	SRP254184	PRJNA615661	SAMN14464290	436	appendix_vermiformis	CP1802v436_GATAGA-C	16S rDNA caecum	AMPLICON	METAGENOMIC	PCR	paired	ILLUMINA	Illumina MiSeq	PCR of V3-V4 DNA16S region	fastq	CP1802v436_GATAGA-CCCBN_L001_R1.fastq.gz	CP1802v436_GATAGA-CCCBN_L001_R2.fastq.gz
SRR11429960	SRP254184	PRJNA615661	SAMN14464291	437	appendix_vermiformis	CP1802v437_TATCAT-C	16S rDNA caecum	AMPLICON	METAGENOMIC	PCR	paired	ILLUMINA	Illumina MiSeq	PCR of V3-V4 DNA16S region	fastq	CP1802v437_TATCAT-CCCBN_L001_R1.fastq.gz	CP1802v437_TATCAT-CCCBN_L001_R2.fastq.gz
SRR11429959	SRP254184	PRJNA615661	SAMN14464292	438	appendix_vermiformis	CP1802v438_CTAGTC-C	16S rDNA caecum	AMPLICON	METAGENOMIC	PCR	paired	ILLUMINA	Illumina MiSeq	PCR of V3-V4 DNA16S region	fastq	CP1802v438_CTAGTC-CCCBN_L001_R1.fastq.gz	CP1802v438_CTAGTC-CCCBN_L001_R2.fastq.gz
SRR11429957	SRP254184	PRJNA615661	SAMN14464293	439	appendix_vermiformis	CP1802v439_GGCTTG-C	16S rDNA caecum	AMPLICON	METAGENOMIC	PCR	paired	ILLUMINA	Illumina MiSeq	PCR of V3-V4 DNA16S region	fastq	CP1802v439_GGCTTG-CCCBN_L001_R1.fastq.gz	CP1802v439_GGCTTG-CCCBN_L001_R2.fastq.gz
SRR11429956	SRP254184	PRJNA615661	SAMN14464294	440	appendix_vermiformis	CP1802v440_CCTCCC-C	16S rDNA caecum	AMPLICON	METAGENOMIC	PCR	paired	ILLUMINA	Illumina MiSeq	PCR of V3-V4 DNA16S region	fastq	CP1802v440_CCTCCC-CCCBN_L001_R1.fastq.gz	CP1802v440_CCTCCC-CCCBN_L001_R2.fastq.gz
SRR11429955	SRP254184	PRJNA615661	SAMN14464295	442	appendix_vermiformis	CP1802v442_AGGGCA-C	16S rDNA caecum	AMPLICON	METAGENOMIC	PCR	paired	ILLUMINA	Illumina MiSeq	PCR of V3-V4 DNA16S region	fastq	CP1802v442_AGGGCA-CCCBN_L001_R1.fastq.gz	CP1802v442_AGGGCA-CCCBN_L001_R2.fastq.gz
SRR11429954	SRP254184	PRJNA615661	SAMN14464296	444	appendix_vermiformis	CP1802v444_GATCTG-C	16S rDNA caecum	AMPLICON	METAGENOMIC	PCR	paired	ILLUMINA	Illumina MiSeq	PCR of V3-V4 DNA16S region	fastq	CP1802v444_GATCTG-CCCBN_L001_R1.fastq.gz	CP1802v444_GATCTG-CCCBN_L001_R2.fastq.gz
SRR11429953	SRP254184	PRJNA615661	SAMN14464297	446	appendix_vermiformis	CP1802v446_GCCGCG-C	16S rDNA caecum	AMPLICON	METAGENOMIC	PCR	paired	ILLUMINA	Illumina MiSeq	PCR of V3-V4 DNA16S region	fastq	CP1802v446_GCCGCG-CCCBN_L001_R1.fastq.gz	CP1802v446_GCCGCG-CCCBN_L001_R2.fastq.gz
SRR11429952	SRP254184	PRJNA615661	SAMN14464298	447	appendix_vermiformis	CP1802v447_TAGGAA-C	16S rDNA caecum	AMPLICON	METAGENOMIC	PCR	paired	ILLUMINA	Illumina MiSeq	PCR of V3-V4 DNA16S region	fastq	CP1802v447_TAGGAA-CCCBN_L001_R1.fastq.gz	CP1802v447_TAGGAA-CCCBN_L001_R2.fastq.gz
SRR11429951	SRP254184	PRJNA615661	SAMN14464299	448	appendix_vermiformis	CP1802v448_TATCGA-C	16S rDNA caecum	AMPLICON	METAGENOMIC	PCR	paired	ILLUMINA	Illumina MiSeq	PCR of V3-V4 DNA16S region	fastq	CP1802v448_TATCGA-CCCBN_L001_R1.fastq.gz	CP1802v448_TATCGA-CCCBN_L001_R2.fastq.gz
SRR11429950	SRP254184	PRJNA615661	SAMN14464300	449	appendix_vermiformis	CP1802v449_TCGAGG-C	16S rDNA caecum	AMPLICON	METAGENOMIC	PCR	paired	ILLUMINA	Illumina MiSeq	PCR of V3-V4 DNA16S region	fastq	CP1802v449_TCGAGG-CCCBN_L001_R1.fastq.gz	CP1802v449_TCGAGG-CCCBN_L001_R2.fastq.gz
SRR11429949	SRP254184	PRJNA615661	SAMN14464301	450	appendix_vermiformis	CP1802v450_CGATAC-C	16S rDNA caecum	AMPLICON	METAGENOMIC	PCR	paired	ILLUMINA	Illumina MiSeq	PCR of V3-V4 DNA16S region	fastq	CP1802v450_CGATAC-CCCBN_L001_R1.fastq.gz	CP1802v450_CGATAC-CCCBN_L001_R2.fastq.gz
SRR11429948	SRP254184	PRJNA615661	SAMN14464302	451	appendix_vermiformis	CP1802v451_TGGTCA-C	16S rDNA caecum	AMPLICON	METAGENOMIC	PCR	paired	ILLUMINA	Illumina MiSeq	PCR of V3-V4 DNA16S region	fastq	CP1802v451_TGGTCA-CCCBN_L001_R1.fastq.gz	CP1802v451_TGGTCA-CCCBN_L001_R2.fastq.gz
SRR11429946	SRP254184	PRJNA615661	SAMN14464303	452	appendix_vermiformis	CP1802v452_CACCGG-C	16S rDNA caecum	AMPLICON	METAGENOMIC	PCR	paired	ILLUMINA	Illumina MiSeq	PCR of V3-V4 DNA16S region	fastq	CP1802v452_CACCGG-CCCBN_L001_R1.fastq.gz	CP1802v452_CACCGG-CCCBN_L001_R2.fastq.gz
SRR11429945	SRP254184	PRJNA615661	SAMN14464304	453	appendix_vermiformis	CP1802v453_TCATGT-C	16S rDNA caecum	AMPLICON	METAGENOMIC	PCR	paired	ILLUMINA	Illumina MiSeq	PCR of V3-V4 DNA16S region	fastq	CP1802v453_TCATGT-CCCBN_L001_R1.fastq.gz	CP1802v453_TCATGT-CCCBN_L001_R2.fastq.gz
SRR11429944	SRP254184	PRJNA615661	SAMN14464305	454	appendix_vermiformis	CP1802v454_TCTCTC-C	16S rDNA caecum	AMPLICON	METAGENOMIC	PCR	paired	ILLUMINA	Illumina MiSeq	PCR of V3-V4 DNA16S region	fastq	CP1802v454_TCTCTC-CCCBN_L001_R1.fastq.gz	CP1802v454_TCTCTC-CCCBN_L001_R2.fastq.gz
SRR11429943	SRP254184	PRJNA615661	SAMN14464306	455	appendix_vermiformis	CP1802v455_GTAGTT-C	16S rDNA caecum	AMPLICON	METAGENOMIC	PCR	paired	ILLUMINA	Illumina MiSeq	PCR of V3-V4 DNA16S region	fastq	CP1802v455_GTAGTT-CCCBN_L001_R1.fastq.gz	CP1802v455_GTAGTT-CCCBN_L001_R2.fastq.gz
SRR11429942	SRP254184	PRJNA615661	SAMN14464307	457	appendix_vermiformis	CP1802v457_AACCGA-C	16S rDNA caecum	AMPLICON	METAGENOMIC	PCR	paired	ILLUMINA	Illumina MiSeq	PCR of V3-V4 DNA16S region	fastq	CP1802v457_AACCGA-CCCBN_L001_R1.fastq.gz	CP1802v457_AACCGA-CCCBN_L001_R2.fastq.gz
SRR11429941	SRP254184	PRJNA615661	SAMN14464308	458	appendix_vermiformis	CP1802v458_GGCAAT-C	16S rDNA caecum	AMPLICON	METAGENOMIC	PCR	paired	ILLUMINA	Illumina MiSeq	PCR of V3-V4 DNA16S region	fastq	CP1802v458_GGCAAT-CCCBN_L001_R1.fastq.gz	CP1802v458_GGCAAT-CCCBN_L001_R2.fastq.gz
SRR11429940	SRP254184	PRJNA615661	SAMN14464309	460	appendix_vermiformis	CP1802v460_CTCATA-C	16S rDNA caecum	AMPLICON	METAGENOMIC	PCR	paired	ILLUMINA	Illumina MiSeq	PCR of V3-V4 DNA16S region	fastq	CP1802v460_CTCATA-CCCBN_L001_R1.fastq.gz	CP1802v460_CTCATA-CCCBN_L001_R2.fastq.gz
SRR11429939	SRP254184	PRJNA615661	SAMN14464310	461	appendix_vermiformis	CP1802v461_GAGTTG-C	16S rDNA caecum	AMPLICON	METAGENOMIC	PCR	paired	ILLUMINA	Illumina MiSeq	PCR of V3-V4 DNA16S region	fastq	CP1802v461_GAGTTG-CCCBN_L001_R1.fastq.gz	CP1802v461_GAGTTG-CCCBN_L001_R2.fastq.gz
SRR11429938	SRP254184	PRJNA615661	SAMN14464311	463	appendix_vermiformis	CP1802v463_AATTCT-C	16S rDNA caecum	AMPLICON	METAGENOMIC	PCR	paired	ILLUMINA	Illumina MiSeq	PCR of V3-V4 DNA16S region	fastq	CP1802v463_AATTCT-CCCBN_L001_R1.fastq.gz	CP1802v463_AATTCT-CCCBN_L001_R2.fastq.gz
SRR11429937	SRP254184	PRJNA615661	SAMN14464312	465	appendix_vermiformis	CP1802v465_CTAGGA-C	16S rDNA caecum	AMPLICON	METAGENOMIC	PCR	paired	ILLUMINA	Illumina MiSeq	PCR of V3-V4 DNA16S region	fastq	CP1802v465_CTAGGA-CCCBN_L001_R1.fastq.gz	CP1802v465_CTAGGA-CCCBN_L001_R2.fastq.gz
SRR11429935	SRP254184	PRJNA615661	SAMN14464313	466	appendix_vermiformis	CP1802v466_TGACCT-C	16S rDNA caecum	AMPLICON	METAGENOMIC	PCR	paired	ILLUMINA	Illumina MiSeq	PCR of V3-V4 DNA16S region	fastq	CP1802v466_TGACCT-CCCBN_L001_R1.fastq.gz	CP1802v466_TGACCT-CCCBN_L001_R2.fastq.gz
SRR11429934	SRP254184	PRJNA615661	SAMN14464314	467	appendix_vermiformis	CP1802v467_CAGTTC-C	16S rDNA caecum	AMPLICON	METAGENOMIC	PCR	paired	ILLUMINA	Illumina MiSeq	PCR of V3-V4 DNA16S region	fastq	CP1802v467_CAGTTC-CCCBN_L001_R1.fastq.gz	CP1802v467_CAGTTC-CCCBN_L001_R2.fastq.gz
SRR11429933	SRP254184	PRJNA615661	SAMN14464315	468	appendix_vermiformis	CP1802v468_TGCAGT-C	16S rDNA caecum	AMPLICON	METAGENOMIC	PCR	paired	ILLUMINA	Illumina MiSeq	PCR of V3-V4 DNA16S region	fastq	CP1802v468_TGCAGT-CCCBN_L001_R1.fastq.gz	CP1802v468_TGCAGT-CCCBN_L001_R2.fastq.gz
SRR11429932	SRP254184	PRJNA615661	SAMN14464316	469	appendix_vermiformis	CP1802v469_AATGAA-C	16S rDNA caecum	AMPLICON	METAGENOMIC	PCR	paired	ILLUMINA	Illumina MiSeq	PCR of V3-V4 DNA16S region	fastq	CP1802v469_AATGAA-CCCBN_L001_R1.fastq.gz	CP1802v469_AATGAA-CCCBN_L001_R2.fastq.gz
SRR11429931	SRP254184	PRJNA615661	SAMN14464317	471	appendix_vermiformis	CP1802v471_CACTGT-C	16S rDNA caecum	AMPLICON	METAGENOMIC	PCR	paired	ILLUMINA	Illumina MiSeq	PCR of V3-V4 DNA16S region	fastq	CP1802v471_CACTGT-CCCBN_L001_R1.fastq.gz	CP1802v471_CACTGT-CCCBN_L001_R2.fastq.gz
SRR11429830	SRP254184	PRJNA615661	SAMN14464318	472	appendix_vermiformis	CP1802v472_GTGCCA-C	16S rDNA caecum	AMPLICON	METAGENOMIC	PCR	paired	ILLUMINA	Illumina MiSeq	PCR of V3-V4 DNA16S region	fastq	CP1802v472_GTGCCA-CCCBN_L001_R1.fastq.gz	CP1802v472_GTGCCA-CCCBN_L001_R2.fastq.gz
SRR11429829	SRP254184	PRJNA615661	SAMN14464319	474	appendix_vermiformis	CP1802v474_GTGACG-C	16S rDNA caecum	AMPLICON	METAGENOMIC	PCR	paired	ILLUMINA	Illumina MiSeq	PCR of V3-V4 DNA16S region	fastq	CP1802v474_GTGACG-CCCBN_L001_R1.fastq.gz	CP1802v474_GTGACG-CCCBN_L001_R2.fastq.gz
SRR11429828	SRP254184	PRJNA615661	SAMN14464320	475	appendix_vermiformis	CP1802v475_GGTCCG-C	16S rDNA caecum	AMPLICON	METAGENOMIC	PCR	paired	ILLUMINA	Illumina MiSeq	PCR of V3-V4 DNA16S region	fastq	CP1802v475_GGTCCG-CCCBN_L001_R1.fastq.gz	CP1802v475_GGTCCG-CCCBN_L001_R2.fastq.gz
SRR11429827	SRP254184	PRJNA615661	SAMN14464321	476	appendix_vermiformis	CP1802v476_AACTTA-C	16S rDNA caecum	AMPLICON	METAGENOMIC	PCR	paired	ILLUMINA	Illumina MiSeq	PCR of V3-V4 DNA16S region	fastq	CP1802v476_AACTTA-CCCBN_L001_R1.fastq.gz	CP1802v476_AACTTA-CCCBN_L001_R2.fastq.gz
SRR11429826	SRP254184	PRJNA615661	SAMN14464322	478	appendix_vermiformis	CP1802v478_CAACGA-C	16S rDNA caecum	AMPLICON	METAGENOMIC	PCR	paired	ILLUMINA	Illumina MiSeq	PCR of V3-V4 DNA16S region	fastq	CP1802v478_CAACGA-CCCBN_L001_R1.fastq.gz	CP1802v478_CAACGA-CCCBN_L001_R2.fastq.gz
SRR11429824	SRP254184	PRJNA615661	SAMN14464323	479	appendix_vermiformis	CP1802v479_AAGGTG-C	16S rDNA caecum	AMPLICON	METAGENOMIC	PCR	paired	ILLUMINA	Illumina MiSeq	PCR of V3-V4 DNA16S region	fastq	CP1802v479_AAGGTG-CCCBN_L001_R1.fastq.gz	CP1802v479_AAGGTG-CCCBN_L001_R2.fastq.gz
SRR11429823	SRP254184	PRJNA615661	SAMN14464324	480	appendix_vermiformis	CP1802v480_TTCAAC-C	16S rDNA caecum	AMPLICON	METAGENOMIC	PCR	paired	ILLUMINA	Illumina MiSeq	PCR of V3-V4 DNA16S region	fastq	CP1802v480_TTCAAC-CCCBN_L001_R1.fastq.gz	CP1802v480_TTCAAC-CCCBN_L001_R2.fastq.gz
SRR11429822	SRP254184	PRJNA615661	SAMN14464325	481	appendix_vermiformis	CP1802v481_ACGAAT-C	16S rDNA caecum	AMPLICON	METAGENOMIC	PCR	paired	ILLUMINA	Illumina MiSeq	PCR of V3-V4 DNA16S region	fastq	CP1802v481_ACGAAT-CCCBN_L001_R1.fastq.gz	CP1802v481_ACGAAT-CCCBN_L001_R2.fastq.gz
SRR11429821	SRP254184	PRJNA615661	SAMN14464326	482	appendix_vermiformis	CP1802v482_GGATTC-C	16S rDNA caecum	AMPLICON	METAGENOMIC	PCR	paired	ILLUMINA	Illumina MiSeq	PCR of V3-V4 DNA16S region	fastq	CP1802v482_GGATTC-CCCBN_L001_R1.fastq.gz	CP1802v482_GGATTC-CCCBN_L001_R2.fastq.gz
SRR11429820	SRP254184	PRJNA615661	SAMN14464327	483	appendix_vermiformis	CP1802v483_CAGGAC-C	16S rDNA caecum	AMPLICON	METAGENOMIC	PCR	paired	ILLUMINA	Illumina MiSeq	PCR of V3-V4 DNA16S region	fastq	CP1802v483_CAGGAC-CCCBN_L001_R1.fastq.gz	CP1802v483_CAGGAC-CCCBN_L001_R2.fastq.gz
SRR11429819	SRP254184	PRJNA615661	SAMN14464328	484	appendix_vermiformis	CP1802v484_GCATGG-C	16S rDNA caecum	AMPLICON	METAGENOMIC	PCR	paired	ILLUMINA	Illumina MiSeq	PCR of V3-V4 DNA16S region	fastq	CP1802v484_GCATGG-CCCBN_L001_R1.fastq.gz	CP1802v484_GCATGG-CCCBN_L001_R2.fastq.gz
SRR11429818	SRP254184	PRJNA615661	SAMN14464329	485	appendix_vermiformis	CP1802v485_CTGCAC-C	16S rDNA caecum	AMPLICON	METAGENOMIC	PCR	paired	ILLUMINA	Illumina MiSeq	PCR of V3-V4 DNA16S region	fastq	CP1802v485_CTGCAC-CCCBN_L001_R1.fastq.gz	CP1802v485_CTGCAC-CCCBN_L001_R2.fastq.gz
SRR11429817	SRP254184	PRJNA615661	SAMN14464330	486	appendix_vermiformis	CP1802v486_TTTCCG-C	16S rDNA caecum	AMPLICON	METAGENOMIC	PCR	paired	ILLUMINA	Illumina MiSeq	PCR of V3-V4 DNA16S region	fastq	CP1802v486_TTTCCG-CCCBN_L001_R1.fastq.gz	CP1802v486_TTTCCG-CCCBN_L001_R2.fastq.gz
SRR11429816	SRP254184	PRJNA615661	SAMN14464331	488	appendix_vermiformis	CP1802v488_AATGGT-C	16S rDNA caecum	AMPLICON	METAGENOMIC	PCR	paired	ILLUMINA	Illumina MiSeq	PCR of V3-V4 DNA16S region	fastq	CP1802v488_AATGGT-CCCBN_L001_R1.fastq.gz	CP1802v488_AATGGT-CCCBN_L001_R2.fastq.gz
SRR11429815	SRP254184	PRJNA615661	SAMN14464332	489	appendix_vermiformis	CP1802v489_GTAACA-C	16S rDNA caecum	AMPLICON	METAGENOMIC	PCR	paired	ILLUMINA	Illumina MiSeq	PCR of V3-V4 DNA16S region	fastq	CP1802v489_GTAACA-CCCBN_L001_R1.fastq.gz	CP1802v489_GTAACA-CCCBN_L001_R2.fastq.gz
SRR11429813	SRP254184	PRJNA615661	SAMN14464333	491	appendix_vermiformis	CP1802v491_CAGGCG-C	16S rDNA caecum	AMPLICON	METAGENOMIC	PCR	paired	ILLUMINA	Illumina MiSeq	PCR of V3-V4 DNA16S region	fastq	CP1802v491_CAGGCG-CCCBN_L001_R1.fastq.gz	CP1802v491_CAGGCG-CCCBN_L001_R2.fastq.gz
SRR11429812	SRP254184	PRJNA615661	SAMN14464334	493	appendix_vermiformis	CP1802v493_ACGGTA-C	16S rDNA caecum	AMPLICON	METAGENOMIC	PCR	paired	ILLUMINA	Illumina MiSeq	PCR of V3-V4 DNA16S region	fastq	CP1802v493_ACGGTA-CCCBN_L001_R1.fastq.gz	CP1802v493_ACGGTA-CCCBN_L001_R2.fastq.gz
SRR11429811	SRP254184	PRJNA615661	SAMN14464335	494	appendix_vermiformis	CP1802v494_ACACCG-C	16S rDNA caecum	AMPLICON	METAGENOMIC	PCR	paired	ILLUMINA	Illumina MiSeq	PCR of V3-V4 DNA16S region	fastq	CP1802v494_ACACCG-CCCBN_L001_R1.fastq.gz	CP1802v494_ACACCG-CCCBN_L001_R2.fastq.gz
SRR11429810	SRP254184	PRJNA615661	SAMN14464336	495	appendix_vermiformis	CP1802v495_AGTATT-C	16S rDNA caecum	AMPLICON	METAGENOMIC	PCR	paired	ILLUMINA	Illumina MiSeq	PCR of V3-V4 DNA16S region	fastq	CP1802v495_AGTATT-CCCBN_L001_R1.fastq.gz	CP1802v495_AGTATT-CCCBN_L001_R2.fastq.gz
SRR11429809	SRP254184	PRJNA615661	SAMN14464337	499	appendix_vermiformis	CP1802v499_CCGGGT-C	16S rDNA caecum	AMPLICON	METAGENOMIC	PCR	paired	ILLUMINA	Illumina MiSeq	PCR of V3-V4 DNA16S region	fastq	CP1802v499_CCGGGT-CCCBN_L001_R1.fastq.gz	CP1802v499_CCGGGT-CCCBN_L001_R2.fastq.gz
SRR11429808	SRP254184	PRJNA615661	SAMN14464338	500	appendix_vermiformis	CP1802v500_GTCATT-C	16S rDNA caecum	AMPLICON	METAGENOMIC	PCR	paired	ILLUMINA	Illumina MiSeq	PCR of V3-V4 DNA16S region	fastq	CP1802v500_GTCATT-CCCBN_L001_R1.fastq.gz	CP1802v500_GTCATT-CCCBN_L001_R2.fastq.gz

## Technical Validation

### Amplicon generation and sequencing

The quality of the Miseq runs was checked internally using PhiX control as recommended by manufacturer. The quality of the stitching procedure was controlled using four bacterial samples (marine strains) that are run routinely in the sequencing facility in parallel to the current samples. Expected proportions of the latter strains were observed in the corresponding samples (Table 3). Further processing of the amplicon reads (pre-cleaning, chimera removal, filtering following quality-filtering strategies guidelines^20^) led to high-quality sequences for further statistical analysis.

**Table 3:** Proportions of four standard bacterial strains within control samples.

Genus	Relative abundance (%)
Enterobacterales Pseudoalteromonadaceae Pseudoalteromonas	46.39 ± 1.27
Bacillales Planococcaceae Bhargavaea	44.19 ± 0.96
Sphingomonadales Sphingomonadaceae Erythrobacter	4.87 ± 0.67
Sphingomonadales Sphingomonadaceae Porphyrobacter	4.47 ± 0.85

Those control samples aimed to validate the metagenomics assembly procedure across the three runs performed (n = 3 with one control sample per run, mean ± sd). The results obtained from 16S rRNA sequencing (Illumina MiSeq) after computational steps showed expected relative abundances.

**Fig. 3:**
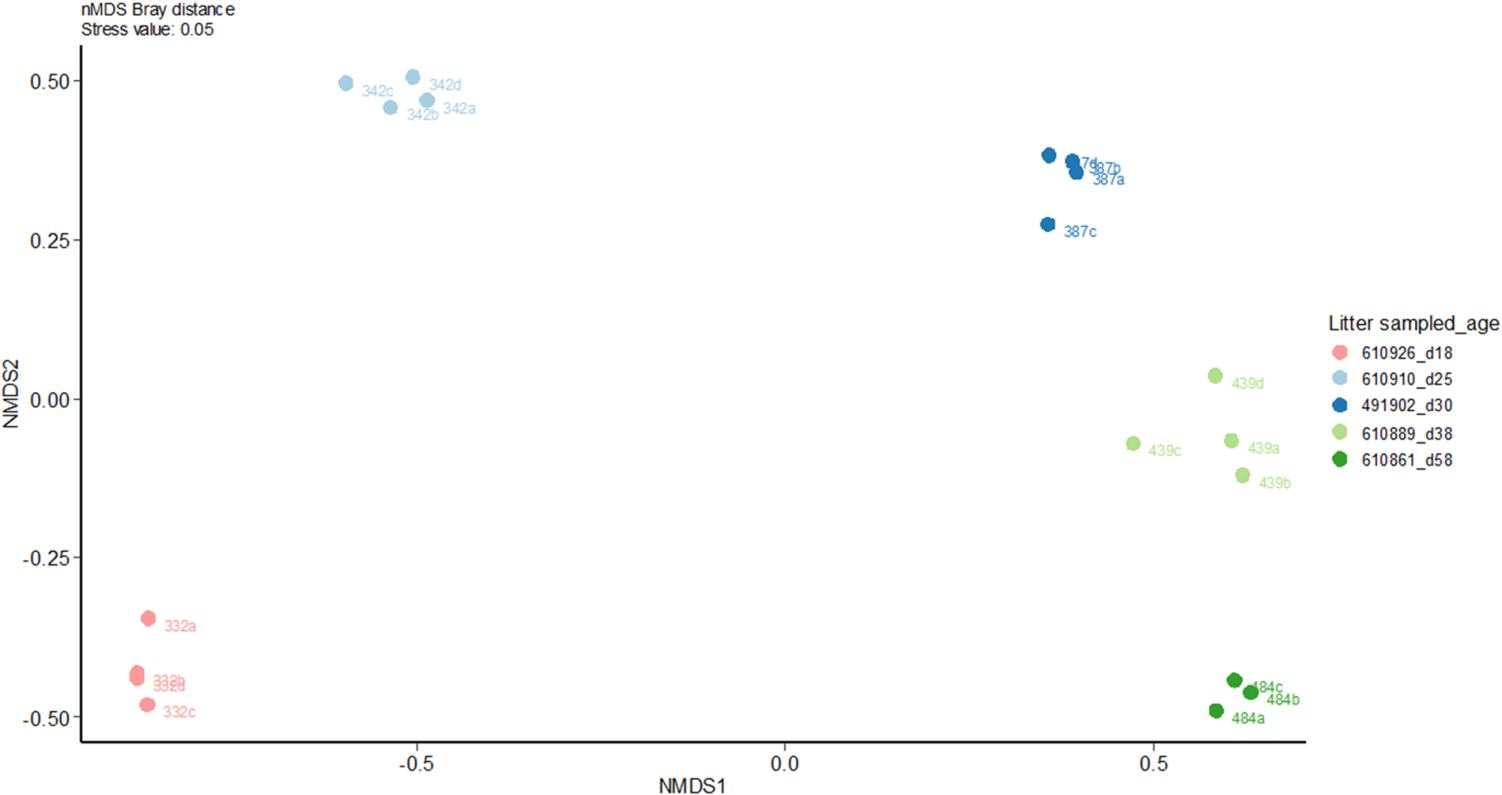
Assessment of microbiota variability due to the DNA extraction step. Bray Curtis dissimilarities were computed using 16S rDNA from caecal contents extracted several time with the same procedure and similar follow-up processing (sequencing and bioinformatics analysis). The numbers refer to the individuals sampled (rabbits of different litters aged from 18 to 58 days) and the letters a, b, c, d refer to the replicates.

Controls for technical reproducibility were performed by assessing the variability due to DNA extraction. Five samples were extracted three or four times in one plate, following similar protocol, and were then sequenced in one MiSeq Illumina Sequencing runs (Supplementary Table S2). Non-metric Multidimensional Scaling (nMDS) projections of Bray-Curtis dissimilarities confirmed limited effect of the extraction protocol on the microbiota structure (Fig. 3).

**Fig. 4:**
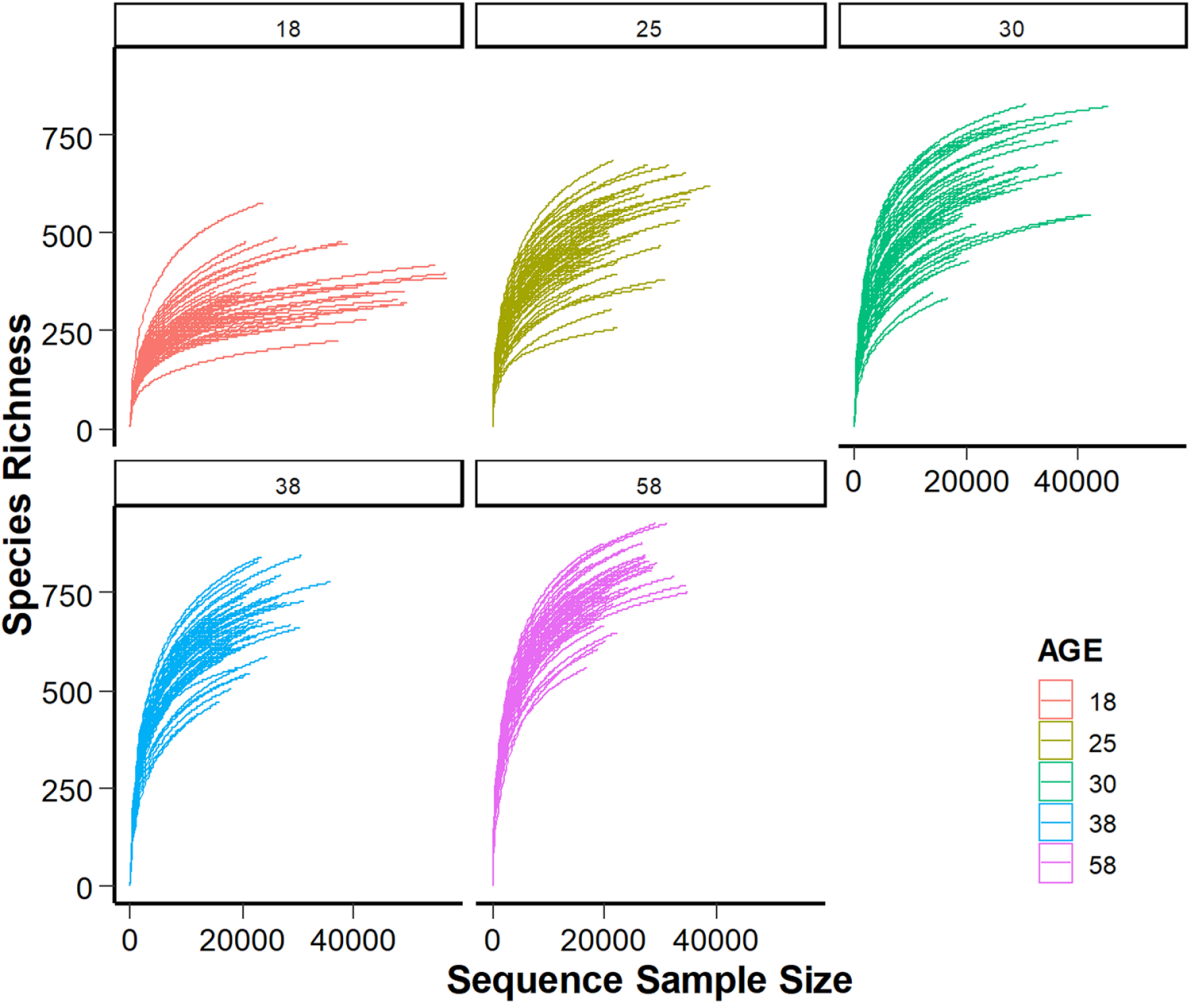
Rarefaction curves for caecal and appendix sites of each individuals (n = 30 individuals per sampling time with two gut sections investigated, i.e. 60 samples on average per age).

Rarefaction curves, constructed for each individual sample, generally approached saturation which indicated sufficient read depth (Fig. 4). Furthermore, rarefaction curves showed different asymptotes according to rabbit physiological development, which was expected given the limited amount of plant polysaccharides in the diets of young suckling rabbits.

### Gene expression profiling

Regarding the high throughput real-time qPCR performed, blank and water controls present in each sample plate allowed us verifying the amplification process per gene. Seven samples were also replicated in each 96-well plate to assess plate-to-plate consistency for caecal and ileal tissue. Based on the Ct values of those samples, we respectively obtained inter-assay coefficients of variation (CV) of 3.0% and 4.0% in the caecum and ileon. The CV of each replicates are specified in Supplemental Fig. 1 for *GAPDH* housekeeping gene and the two genes whose expression was significantly modulated by one of the experimental treatments^12^.

### Blood nutritionnal status

Biochemical measurements of blood content were performed on two batches with a repeated control of known concentration (human serum) and a repeated pool of samples (two repetitions per batch). The control measurements fell within appropriate confidence limits and the CV values for the pool repetitions were between 2 and 4% thus ensuring comparable results between batches for the three analytes measured.

### Supplementary information

Supplemental table S1

Supplemental table S2

Supplemental Figure 1

## Data Availability

The workflow for data analysis using R version 4.0.0 is proposed at this address: https://github.com/paescharlotte/early_life_nutrition_rabbit/^17^.
