# Supplementary material for: A multi-omics dataset of the response to early plant polysaccharide ingestion in rabbits
Source: Sci Data. 2024 Jun 25;11:684. doi: 10.1038/s41597-024-03471-1 (PMC11199578; doi:10.1038/s41597-024-03471-1)
Supplement: Supplementary file 1 — Supplemental table S1 [file 41597_2024_3471_MOESM1_ESM.doc]

**Supplementary Information**

Supplemental table S1: **Identification of metabolites in caecal content NMR spectra**. s: singulet, d: doublet, t: triplet, q: quintuplet; m: multiplet, *: indicate the peak used for quantification.

|  | Metabolite | δ^1^H (ppm) |
| --- | --- | --- |
| 1 | Butyrate | 0.90* (t), 1.56 (m), 2.16 (t) |
| 2 | 4-methyl-2-oxovalerate | 0.94 (d), 2.62* (d) |
| 3 | Isoleucine | 0.94 (t), 1.01* (d) |
| 4 | Leucine | 0.97* (t) |
| 5 | Valine | 1.00* (d), 1.05 (d) |
| 6 | Propionate | 1.06* (t), 2.19 (q) |
| 7 | 3-methyl-2-oxovalerate | 1.10* (d) |
| 8 | 3-methyl-2-oxobutyrate | 1.13* (d) |
| 9 | Ethanol | 1.18* (t) |
| 10 | Valerate | 0.89 (t), 1.30* (m), 1.53 (m), 2.19 (t) |
| 11 | Threonine | 1.34* (d), 3.60 (d), 4.26 (m) |
| 12 | Lysine | 1.74* (m), 3.03 (t), 3.77 (t) |
| 13 | Acetate | 1.92 *(s) |
| 14 | Glutamate | 2.08 (m), 2.35* (m) |
| 15 | Succinate | 2.41* (s) |
| 16 | 3-(3-hydroxyphenyl)propionate | 2.48 (t), 2.85 (t), 6.76 (d), 6.80* (s), 6.87 (d), 7.25 (t) |
| 17 | Methylamine | 2.60* (s) |
| 18 | Dimethylamine | 2.72* (s) |
| 19 | Trimethylamine | 2.89* (s) |
| 20 | 3-phenylpropionate | 2.50 (t), 2.90* (t), 7.27 (t), 7.32 (d), 7.37 (t) |
| 21 | Choline | 3.21* (s) |
| 22 | Glucose | 3.25 (t), 3.40 – 3.56 (m), 3.71 – 3.91 (m), 5.24* (d) |
| 23 | Methanol | 3.36* (s) |
| 24 | Glycine | 3.57* (s) |
| 25 | Ribose | 5.26 (s), 5.39* (d) |
| 26 | Galactose | 5.27* (d) |
| 27 | Uracil | 5.81* (d), 7.55 (d) |
| 28 | Tyrosine | 6.91 (d), 7.20* (d) |
| 29 | Phenyalanine | 7.33 (d), 7.38 (t), 7.43* (t) |
