# Supplementary figures and images for: A multi-omics dataset of the response to early plant polysaccharide ingestion in rabbits
Source: Sci Data. 2024 Jun 25;11:684. doi: 10.1038/s41597-024-03471-1 (PMC11199578; doi:10.1038/s41597-024-03471-1)

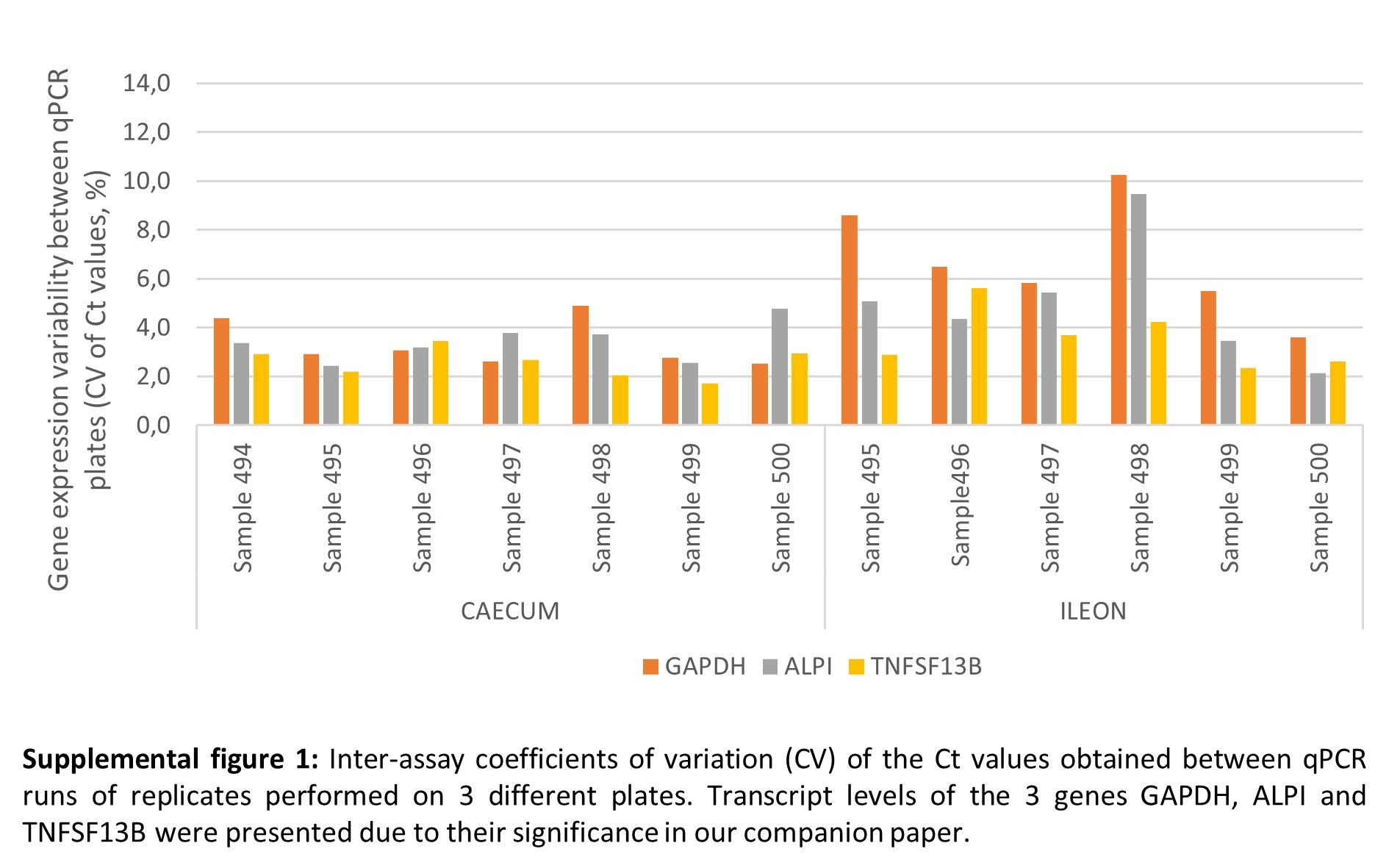

Supplement: Supplementary file 3 — Supplemental Figure 1 [file 41597_2024_3471_MOESM3_ESM.tif]
